# Chromosomes in the flow to simplify genome analysis

**DOI:** 10.1007/s10142-012-0293-0

**Published:** 2012-08-16

**Authors:** Jaroslav Doležel, Jan Vrána, Jan Šafář, Jan Bartoš, Marie Kubaláková, Hana Šimková

**Affiliations:** Centre of the Region Haná for Biotechnological and Agricultural Research, Institute of Experimental Botany, Sokolovská 6, 77200 Olomouc, Czech Republic

**Keywords:** Chromosome sorting, Chromosome-specific BAC libraries, Chromosome sequencing, Chromosome genomics, Genome complexity reduction, Flow cytometry, Physical mapping

## Abstract

Nuclear genomes of human, animals, and plants are organized into subunits called chromosomes. When isolated into aqueous suspension, mitotic chromosomes can be classified using flow cytometry according to light scatter and fluorescence parameters. Chromosomes of interest can be purified by flow sorting if they can be resolved from other chromosomes in a karyotype. The analysis and sorting are carried out at rates of 10^2^–10^4^ chromosomes per second, and for complex genomes such as wheat the flow sorting technology has been ground-breaking in reducing genome complexity for genome sequencing. The high sample rate provides an attractive approach for karyotype analysis (flow karyotyping) and the purification of chromosomes in large numbers. In characterizing the chromosome complement of an organism, the high number that can be studied using flow cytometry allows for a statistically accurate analysis. Chromosome sorting plays a particularly important role in the analysis of nuclear genome structure and the analysis of particular and aberrant chromosomes. Other attractive but not well-explored features include the analysis of chromosomal proteins, chromosome ultrastructure, and high-resolution mapping using FISH. Recent results demonstrate that chromosome flow sorting can be coupled seamlessly with DNA array and next-generation sequencing technologies for high-throughput analyses. The main advantages are targeting the analysis to a genome region of interest and a significant reduction in sample complexity. As flow sorters can also sort single copies of chromosomes, shotgun sequencing DNA amplified from them enables the production of haplotype-resolved genome sequences. This review explains the principles of flow cytometric chromosome analysis and sorting (flow cytogenetics), discusses the major uses of this technology in genome analysis, and outlines future directions.

## Introduction

With some rare exceptions (Crosland and Crozier 1986), nuclear genomes of animals and plants are made not of a single but several molecules of DNA, each of them forming a highly organized structure called chromosome. Chromosomes are formed by packing DNA into a small space via the interaction with histones and non-histone proteins, which also play an important role in the regulation of gene expression (Margueron and Reinberg 2010; Zhou et al. 2011). Although there is no obvious relationship between genome size and the number of chromosomes (Heslop-Harrison and Schwarzacher 2011), it is believed that there is an upper limit of chromosome size and large genomes must be distributed into several smaller chromosomes (Schubert and Oud 1997). The interaction of kinetochore structures formed on chromosomes with the spindle apparatus ensures an ordered separation of replicated DNA into daughter cells during mitosis (Verdaasdonk and Bloom 2011; Gordon et al. 2012) and production of functional gametes during meiosis (Brar and Amon 2008; Pawlowski 2010). Recombination and random segregation of homologous chromosomes during meiosis is crucial for generating genetic variation (Yanowitz 2010; Lichten and de Massy 2011; Osman et al. 2011).

Genome mapping, sequencing, and gene isolation projects have, to date, rarely exploited the organization of plant and animal genomes into the chromosomes. Yet, plant and animal genomes may be large and complex because of a high content of repetitive and duplicated DNA sequences. The complexity of some plant genomes is further augmented by allopolyploidy resulting from the presence of two or more structurally similar chromosome sets originating from different parental species. These features hamper the construction of clone-based physical maps, positional gene cloning, and de novo genome sequencing. Although it is not a problem to fingerprint the large numbers of clones needed to establish a physical map (Luo et al. 2003), and to sequence billions of DNA bases (Metzker 2010), the difficulty is to arrange the large number of fingerprints and short reads into an unambiguous order that faithfully represents the genome (Wei et al. 2009; Alkan et al. 2011; Treangen and Salzberg 2012). Another area which profits from the analysis at single-chromosome level is the production of haplotype-resolved genome sequences (Yang et al. 2011).

In this review, we describe how laser flow cytometry can be used to dissect nuclear genomes into single chromosomes in order to provide a basis for a chromosome-focused analysis of the genome. We provide examples of the use of flow-sorted chromosomes to analyze genomes of human, animals, and plants. We conclude that flow cytogenetics holds the key to tackling complex genomes by greatly reducing genome complexity for targeted and cost-effective studies.

## Chromosome isolation and flow cytogenetics

A majority of cells in plant and animal bodies are at interphase and their nuclei contain decondensed chromosomes, which cannot be physically separated from each other. This is possible only during the metaphase stage of cell division when the chromosomes are condensed. Early studies isolated single chromosomes from metaphase spreads of dividing cells using a micromanipulator (Chambers and Sands 1923; Barigozzi 1939; Scalenghe et al. 1981; Schondelmaier et al. 1993). Following the first generation of mechanical micromanipulators, computer-aided instruments using laser technology were developed (Matsunaga et al. 1999). A clear advantage of micromanipulation is that the operator visually identifies chromosomes to be isolated. A disadvantage is that only a small number of chromosomes can be collected (Hobza and Vyskot 2007) and that the quality of chromosomal DNA may be suitable only for some types of analysis (Ma et al. 2010). Recent improvements in the area of chromosome micromanipulation include the atomic force microscope nanolithography, which enables dissection of fragments as small as 0.4 μm (Di Bucchianico et al. 2011).

Isolation of chromosomes from populations of dividing cells into aqueous suspension provides other options for manipulation and the opportunity to isolate chromosomes in large numbers. Separation of chromosomes based on relative density by gradient centrifugation enables the separation of small and large chromosomes only and is therefore not suitable for the isolation of particular chromosomes (Stubblefield and Oro 1982). Another option is to separate a specific chromosome using magnetic beads after hybridization with a biotin- or fluorochrome-labeled chromosome-specific probe (Dudin et al. 1988; Vitharana and Wilson 2006). This approach, however, suffers from lower purities in isolated fractions.

**Fig. 1:**
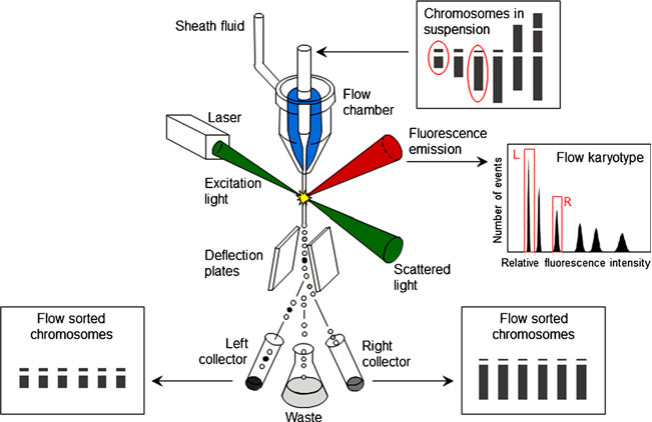
Schematic view of a flow cytometer and sorter. In this example, the instrument is equipped with one laser used as light source. The sample (suspension of intact chromosomes) is stained by a DNA-specific dye and introduced to a flow chamber from which a narrow stream of liquid emerges and carries the chromosomes. The chromosomes in the stream interact individually with the laser beam, and scattered light and emitted fluorescence are quantified. Histogram of fluorescence intensity (flow karyotype) is generated during the analysis and if the chromosome of interest differs in relative fluorescence intensity, it forms a separate peak on flow karyotype and the chromosome can be sorted. Sorting is achieved by breaking the liquid stream into droplets and by electrically charging droplets carrying chromosomes of interest. Chromosome droplets are deflected during a passage through electrostatic field and collected in suitable containers (reproduced from: Meksem and Kahl (2005) with permission)

To date, the most successful and almost universally used approach for separating chromosomes on a preparative scale has been flow cytometry, a method designed to analyze the optical parameters of microscopic particles during a passage in a narrow stream of liquid. Flow cytometry analyzes cells and cell organelles at rates of 10^2^–10^4^/s (Fig. 1). If a chromosome of interest can be resolved from other chromosomes based on its optical properties (light scatter, fluorescence), it can be purified in large quantities.

Flow cytometry to analyze and sort metaphase chromosome (flow cytogenetics, Gray 1989; Bartholdi 1990) is usually applied to mitotic preparations since the preparation of suspensions of meiotic chromosomes is not practical. Tissues and cell cultures from which the samples are prepared must be synchronized to achieve a high proportion of cells in metaphase. In animal systems, up to 95 % of cells can be accumulated in metaphase (Carrano et al. 1976, 1979); a lower degree of synchrony of about 50 % metaphase cells has been achieved in plants (Vrána et al. 2000; Vláčilová et al. 2002). Chromosomes are released from cell populations highly enriched for metaphase cells into a suitable isolation buffer; chromosomal DNA is stained using a DNA-specific fluorochrome to allow chromosomes to be classified according to fluorescence intensity (relative DNA content). The analysis of large populations can be carried out in a short time and results in representative distributions of chromosomal DNA content termed flow karyotypes. Ideally, each chromosome forms a distinct peak on the flow karyotype, whose location is proportional to fluorescence intensity and whose volume is proportional to the frequency of occurrence of that chromosome type. However, due to similarity in size and relative DNA content, peaks of some chromosome types may overlap and the chromosomes cannot be resolved.

## Flow analysis of human and animal chromosomes

**Fig. 2:**
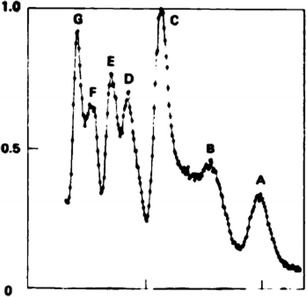
Distribution of relative fluorescence intensity (flow karyotype) of mitotic chromosomes extracted from a human diploid cell strain (2*n* = 46, XY), stained by ethidium bromide and measured in the Livermore flow microfluorometer. Experimental data points are connected by a *solid line*. Seven groups of chromosomes were discriminated, represented by peaks labeled *A*–*G*. *X axis*, relative fluorescence intensity; *Y axis*, frequency of particles (Gray et al. (1975b), with permission, modified)

In their pioneering experiments, Gray et al. (1975a) and Stubblefield et al. (1975) analyzed chromosome suspensions prepared from Chinese hamster cell lines after staining with a DNA fluorochrome, ethidium bromide. Large numbers of chromosomes (10^5^–10^6^) were analyzed in a short time and the position of peaks in flow karyotypes corresponded with the expected distribution of chromosomal DNA content. Microscopic analysis of particles sorted onto microscope slides from each peak showed less than 20 % contamination with other chromosomes. Subsequent flow measurement of chromosomes from two Chinese hamster cell lines indicated a potential to detect chromosome rearrangements such as translocations (Gray et al. 1975b). Initial analysis of human chromosomes obtained from male cell line resulted in a flow karyotype with seven peaks for the 24 chromosome types (Fig. 2; Gray et al. 1975b) and the classification of chromosomes from male deer Indian muntjac according to DNA content led to flow karyotype with five major peaks corresponding to five chromosome types in this species (Carrano et al. 1976). The latter work demonstrated a feasibility to sort chromosomes at rates of many hundreds per second with a purity of 90 % and hence a possibility to collect microgram quantities of purified chromosomes.

**Fig. 3:**
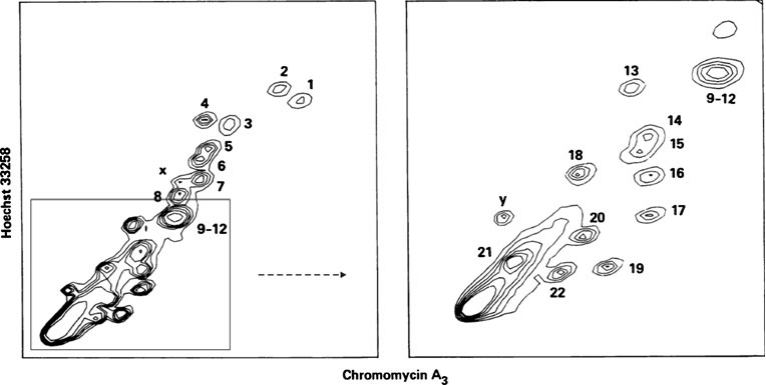
Bivariate flow karyotype of human lymphocyte chromosomes, which were stained with Hoechst 33258 and chromomycin A3. The height of each peak in the distribution is indicated by contours. *Left*, chromosomes 9–12 form a composite peak and cannot be sorted individually. *Right*, expanded view of chromosomes smaller than chromosome 8; chromosomes 14–15 are not clearly resolved. *X axis*, relative fluorescence intensity of chromomycin A3; *Y axis*, relative fluorescence intensity of Hoechst 33258 (Langlois et al. (1982), with permission)

These historical experiments laid the foundations of flow cytogenetics and indicated its potential to characterize karyotypes, identify structural chromosome changes, and purify large quantities of chromosomes for biochemical and molecular analyses. A prerequisite was to improve the resolution of flow karyotyping to increase the number of chromosome types, which can be identified and sorted, and to develop flow cytogenetics in other species. By replacing ethidium bromide with Hoechst 33258 to stain chromosomal DNA, Carrano et al. (1979) improved the resolution of human flow karyotypes so that they could classify the 24 chromosome types into 15 groups. A major advance was made by Langlois et al. (1982) who took the advantage of the differences in AT/GC content among the human chromosomes and analyzed chromosomes stained by two dyes differing in base specificity—Hoechst 33258 (preferential AT-binding) and chromomycin A3 (preferential GC-binding). This so called bivariate flow karyotyping enabled the discrimination of all human chromosomes except chromosomes 9–12 and chromosomes 14 and 15 (Fig. 3). The variability in peak position within the flow karyotype was small enough to detect chromosome gains and losses equivalent to 1/600 genome, and this facilitated the detection of chromosome polymorphism.

Sample quality determines the success in discriminating individual chromosomes and thus the protocols for the preparation of aqueous suspensions of chromosomes must be optimized to maintain chromosome morphology and to ensure that the suspensions are free of cell and chromosomal debris, chromatids, and chromosome clumps (Sillar and Young 1981; Bijman 1983; van den Engh et al. 1984; Aten et al. 1987a; Telenius et al. 1993; Ng and Carter 2006). Improved methods become available to prepare samples not only from cell lines (van den Engh et al. 1984) and peripheral lymphocytes (Matsson and Rydberg 1981; Young et al. 1981) but also from solid tissues and tumors (Kooi et al. 1984). Optimization of chromosome isolation and staining protocols permitted the discrimination and sorting of all human chromosomes except chromosomes 9, 10, 11, and 12 (Gray and Cram 1990). Bivariate flow karyotyping has become a golden standard in human and animals, where it has been used in a number of species (for a list of examples, see, e.g., Ferguson-Smith 1997). However, as in human, not all chromosomes can be resolved in all animal species. In a male dog, the 76 autosomes and two sex chromosomes were resolved into 32 peaks (Langford et al. 1996), and 19 chromosomal pairs of the swine karyotype were resolved into 18 peaks (Yerle et al. 1993). Out of the 78 chromosomes of domestic chicken, only macrochromosomes 1–9, Z and W chromosomes, and three microchromosomes were distinguishable (Nie et al. 2009).

## Plants are different

Only a decade after the first reports in Chinese hamster and human, de Laat and Blaas (1984) reported on flow karyotyping in a plant, *Haplopappus gracilis*, and sorting its two chromosome types. The progress in plants was slow due to a low degree of metaphase synchrony and difficulties with chromosome release from cells with rigid walls (for a review, see Doležel et al. (1994)). Initially, chromosome samples were prepared from suspension cultured cells (de Laat and Blaas 1984; Arumuganathan et al. 1991; Wang et al. 1992). This approach has been abandoned as the cultures are often heterogeneous and difficult to synchronize (Arumuganathan et al. 1991), karyologically unstable (Leitch et al. 1993; Schwarzacher et al. 1997), and not easy to initiate in some species. Conia et al. (1987, 1989) suggested using leaf mesophyll protoplast cultures as an alternative. But as mitotic synchrony induced by transferring isolated protoplasts to nutrient medium was rather low, and as protoplast cultures are hard to establish in many plants, the system has not been adopted by others. Currently the only method used widely describes the preparation of chromosome samples from root tip meristems of young seedlings (Doležel et al. 1992). The meristems are karyologically stable and their cells are easy to synchronize. Some authors used genetically transformed “hairy” root cultures when working with lines that cannot be maintained by seed propagation (Veuskens et al. 1995; Neumann et al. 1998).

**Fig. 4:**
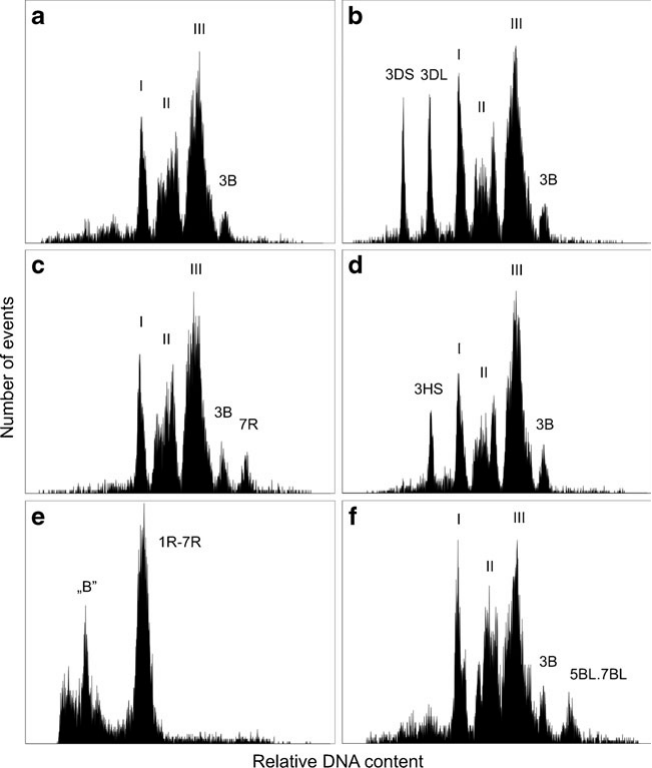
Examples of flow karyotyping in various cytogenetic stocks in plants. Mitotic metaphase chromosomes were isolated from synchronized root tip meristems and stained by DAPI prior to analysis. **a** Flow karyotype of hexaploid wheat (2*n* = 6*x* = 42) comprises three composite peaks representing groups of chromosomes and a peak representing chromosome 3B. Only this chromosome can be sorted from wild-type karyotype. **b** The analysis of a double ditelosomic line dDt3D of wheat in which the arms of chromosome 3D are stably maintained as telocentric chromosomes 3DS and 3DL. The arms are smaller than the remaining chromosomes, are represented by well discriminated peaks on the flow karyotype, and can be easily sorted. **c** Flow karyotype of wheat–rye chromosome addition line 7R comprises peaks representing chromosomes of wheat (*I*–*III* and *3B*) and a peak of chromosome 7R, which can be sorted. **d** The analysis of chromosomes isolated from wheat–barley chromosome arm addition line 3HS results in flow karyotype with a well-discriminated peak of 3HS, which facilitates its sorting. **e** A flow karyotype of rye cv. Adams carrying supernumerary B chromosomes (2*n* = 14 + B) comprises one composite peak representing all rye chromosomes 1R-7R and a peak of chromosome B. **f** Wheat cultivar Arina carries a translocation chromosome 5BL.7BL, which is the largest in the karyotype and is represented by a peak to the right of chromosome 3B

Plant mitotic chromosomes were initially released by lyzing synchronized cells into a hypotonic buffer after the enzymatic removal of their walls (de Laat and Blaas 1984; Arumuganathan et al. 1991; Wang et al. 1992; Veuskens et al. 1995). The method did not work well with root meristems and an alternative method was developed in which the chromosomes were released by mechanical homogenization of formaldehyde-fixed tissues (Doležel et al. 1992; Gualberti et al. 1996). While bivariate flow karyotyping marked a great improvement in flow cytogenetics of human and animals, it did not bring any significant improvement in plants (Lee et al. 1997, 2000; Lucretti and Doležel 1997). The failure was most probably due to the presence of homogenously dispersed repetitive DNA sequences in plants (Fuchs et al. 1996; Schubert et al. 2001). As a result, flow cytometric analysis and sorting is carried out after staining the samples with only one DNA fluorochrome, typically DAPI (Vláčilová et al. 2002; Überall et al. 2004; Kubaláková et al. 2005). The number of chromosomes which can be discriminated varies between species (Table 1; Doležel et al. 2004). For example, only one out of the 21 chromosomes of bread wheat can be discriminated from a wild-type karyotype (Fig. 4a; Vrána et al. 2000), while five out of eight chromosomes can be resolved in chickpea (Vláčilová et al. 2002).

**Table 1:** List of plant species from which flow cytometric analysis of mitotic chromosomes has been reported

Species		Chromosome number (*n*)^a^	Number of discriminated chromosomes		References
Latin name	Common name		Standard karyotype^b^	Cytogenetic stock^c^	
*Aegilops biuncialis*	Goatgrass	14	2	–	Molnár et al. (2011)
*Aegilops comosa*	Goatgrass	7	1	–	Molnár et al. (2011)
*Aegilops umbellulata*	Goatgrass	7	4	–	Molnár et al. (2011)
*Avena sativa*	Oats	21	0	–	Li et al. (2001)
*Cicer arietinum*	Chickpea	8	5	–	Vláčilová et al. (2002) ; Zatloukalová et al. (2011)
*Festuca pratensis*	Meadow fescue	7	1	–	Kopecký et al. (2011)
*Haplopappus gracilis*		2	2	–	de Laat and Blaas (1984); de Laat and Schel (1986)
*Hordeum vulgare*	Barley	7	1 (2)	7	Lysák et al. (1999); Lee et al. (2000); Suchánková et al. (2006)
*Lycopersicon esculentum*	Tomato	12	0	–	Arumuganathan et al. (1991)
*Lycopersicon pennellii*	Tomato	12	2	–	Arumuganathan et al. (1991, 1994)
*Melandrium album*; *Silene latifolia*	White Campion	12	2	–	Veuskens et al. (1995); Kejnovský et al*.* (2001)
*Nicotiana plumbaginifolia*	Tobacco	10	0	–	Conia et al. (1989)
*Oryza sativa*	Rice	12	0	–	Lee and Arumuganathan (1999)
*Petunia hybrida*		7	1	–	Conia et al. (1987)
*Picea abies*	Norway spruce	12	3	–	Überall et al. (2004)
*Pisum sativum*	Pea	7	2	4	Gualberti et al. (1996); Neumann et al. (1998, 2002)
*Secale cereale*	Rye	7	1	7^d^	Kubaláková et al. (2003)
*Triticum aestivum*	Bread wheat	21	1 (2)	21^e^	Wang et al. (1992) ; Schwarzacher et al. (1997) ; Lee et al. (1997) ; Gill et al. (1999); Vrána et al. (2000); Kubaláková et al. (2002)
*Triticum durum*	Durum wheat	14	1	14^f^	Kubaláková et al. (2005)
*Vicia faba*	Field bean	6	1	6	Lucretti et al. (1993); Doležel and Lucretti (1995); Lucretti and Doležel (1997); Kovářová et al. (2007)
*Zea mays*	Maize	10	2 (3)	10^g^	Lee et al. (1996, 2002); Li et al. (2001, 2004)

^a^Number of chromosomes in a haploid set

^b^Number of chromosomes that could be discriminated unambiguously. The numbers in brackets indicate the number of chromosomes that could be discriminated in some lines due to chromosome polymorphism

^c^Number of individual chromosome types discriminated in different lines (translocation, deletion, or addition lines). Note that in some species this option has not been verified

^d^Rye chromosomes 2R–7R could be discriminated from wheat–rye chromosome addition lines (Kubaláková et al. 2003)

^e^Sorting of almost all chromosome arms is possible in hexaploid wheat using individual (di)telosomic lines (Kubaláková et al. 2002)

^f^All chromosome arms may be sorted from individual (di)telosomic lines (Kubaláková et al. 2003)

^g^Oat–maize chromosome addition lines (Li et al. 2001)

## Chromosome characterization by flow

Flow karyotyping is a quantitative, statistically accurate, and high-throughput approach for karyotype analysis and the detection of numerical and structural chromosome changes. Typically 20,000–100,000 chromosomes (in human representing a combined karyotype of at least 400 cells) are analyzed in a short time to generate univariate or bivariate flow karyotypes. This provides an accurate measurement of the frequency of different chromosome types. For instance, trisomy 21 appears as a 50-% increase in the volume of peak representing chromosome 21 as compared with other chromosome types (Gray et al. 1986), and translocations resulting in derivative chromosomes that differ either in DNA content or base pair ratio will appear as new peaks (Lebo et al. 1986). Chromosome fluorescence can be measured with coefficients of variation as low as 1 %, and the size of small deletions can be estimated (Trask et al. 1996). However, flow cytometry is not suitable for karyotype analysis in heterogenous populations. To cope with this limitation, Stepanov et al. (1996) designed a modified flow chamber in which cells are ruptured individually and batches of chromosomes from individual cells are analyzed separately at rates of 10^2^ cells/min. However, to our knowledge, this system has not been adopted by others.

In biomedical research, flow karyotyping has been used to identify translocation chromosomes in pig (Hausmann et al. 1993) and identify its chromosomes in pig–mouse somatic cell hybrid line (Bouvet et al. 1993), analyze karyotype instability during a neoplastic process (Cram et al. 1983), identify tumor marker chromosomes (Nusse et al. 1992), and detect radiation damage (Fantes et al. 1983; Aten et al. 1987b). In plants, flow karyotyping was found to be sensitive enough to detect trisomy of chromosome 6 in barley (Lee et al. 2000) and estimate the frequency of alien chromosomes in populations of six wheat–rye chromosome addition lines (Kubaláková et al. 2003). Translocation chromosomes were identified in field bean, garden pea, barley, and wheat (Fig. 4f) (Doležel and Lucretti 1995; Neumann et al. 1998; Lysák et al. 1999; Vrána et al. 2000; Kubaláková et al. 2002, 2003), and chromosome deletions were investigated in wheat (Gill et al. 1999; Kubaláková et al. 2002, 2005). Accessory B chromosomes were readily detected in rye (Fig. 4e) (Kubaláková et al. 2003) and maize (unpublished observation). Alien chromosomes were identified in oat–maize and wheat–rye chromosome addition lines (Li et al. 2001; Kubaláková et al. 2003); alien chromosome arms were identified in wheat–rye and wheat–barley telosome addition lines (Suchánková et al. 2006; Šimková et al. 2008) and chromosome polymorphism was observed in barley, maize, rye, and wheat (Lee et al. 2000, 2002; Vrána et al. 2000; Kubaláková et al. 2002, 2003).

As the flow karyotyping is based on chromosome DNA content and/or AT/GC ratio, intrachromosomal rearrangements and reciprocal translocations where equal amounts of DNA are exchanged cannot be identified. Detection of aberrations is also hampered by natural occurrence of chromosome polymorphisms (Harris et al. 1986). With these limitations, the labor-intensive character, and a need for expensive equipment, flow karyotyping cannot compete with advanced methods of cytogenetics such as multicolor fluorescence in situ hybridization (FISH), chromosome painting, and DNA arrays. Thus, early hopes that flow karyotyping will be used for semi-automatic detection of aberrant chromosomes (Boschman et al. 1992) have remained unfulfilled. However, in the following, we will show that many of the advanced cytogenetic methods actually rely on using DNA from flow-sorted chromosomes.

## Chromosome sorting principles

The most frequent flow sorter design relies on breaking the narrow liquid jet carrying the microscopic particles of the sample into small droplets in a regular fashion. Droplets carrying chromosomes of interest are charged electrically and deflected during a passage through an electrostatic field (Fig. 1). Like the analysis, sorting can be done at high speeds of up to several hundred chromosomes per second (Gray and Cram 1990). The utility of sorted chromosome fractions is determined by their purity and quality of DNA (or proteins if they are analyzed). Purity depends on the degree to which the chromosome of interest can be resolved from other chromosomes, chromosome clumps, chromatids, and chromosome fragments in the sample. Hence, the sample quality and instrument resolution are critical.

The extent of contamination in sorted fractions has been estimated by microscopic observation of chromosomes sorted onto microscopic slides and subjected either to G-banding (Rommel et al. 1988; Hausmann et al. 1993), N-banding (Gill et al. 1999), and/or FISH with probes, resulting in chromosome-specific labeling pattern (Rommel et al. 1988; VanDevanter et al. 1994; Schmitz et al. 1995; Kubaláková et al. 2003). If alien chromosomes are sorted from a host organism, they may be identified using genomic in situ hybridization with a labeled whole-genome probe (Li et al. 2001). Sorted chromosomes were also identified using polymerase chain reaction (PCR) in situ which does not require labeled probes (Gualberti et al. 1996; Lysák et al. 1999). The ability to identify chromosomes in sorted fractions has been important to characterize flow karyotypes and assign peaks on flow karyotypes to individual chromosomes. Although this can also be done using PCR with chromosome-specific primers (Shepel et al. 1994; Lysák et al. 1999; Vláčilová et al. 2002), microscopic analyses of sorted fractions are preferred as they enable the identification of contaminating particles and determine their frequency (Kubaláková et al. 2000). If sorting is carried out under favorable conditions and at a low sample rate, favoring the high resolution of chromosome peaks, purities higher than 95 % can be achieved (Cram et al. 2002; Mayer et al. 2011).

## Sorting chromosomes that cannot be resolved

The application of flow-sorted chromosomes in genetics and genomics of most of animal and plant species was hindered by the inability to resolve all chromosomes (Fig. 4a). Various approaches have been developed to deal with this and can be classified into two groups: (a) improvements in instrumentation and methodology and (b) judicious selection of genotypes from which desired chromosomes are purified. The efforts to improve the instrumentation lead to development of slit-scan flow cytometry, which classifies chromosomes according to the distribution of fluorescence along their length (Bartholdi et al. 1990; Rens et al. 1994). As the staining intensity is reduced at the centromere, this approach provided the information on centromere position (centromeric index) and the number of centromeres. Bartholdi et al. (1989) demonstrated that chromosome banding may provide additional landmarks along chromosomes. Despite these encouraging observations, slit-scan flow karyotyping did not find a wider use.

The attempts to improve the methodology included immunofluorescent staining of chromosomal proteins. Initial experiments confirmed a possibility to label the proteins of chromosomes in suspension with fluorescent antibodies (Trask et al. 1984). While Fantes et al. (1989) failed to identify dicentric chromosomes induced by radiation after labeling centromeric regions with CREST antibodies, Levy et al. (1991) succeeded in resolving chromosomes 2 and X in Indian muntjac after immunofluorescent antikinetochore staining. Also, this approach has not been followed by others. In principle, labeling of particular DNA sequences should provide a general approach to identify any chromosome. After Trask et al. (1985) succeeded in labeling a specific DNA sequence using FISH on interphase nuclei in suspension and quantifying bound probe by flow cytometry, Dudin et al. (1987) successfully applied the same method to chromosomes in suspension. They used human genomic DNA as probe for FISH to chromosomes isolated from Chinese hamster × human hybrid cell line. However, they did not analyze the chromosomes by flow cytometry. Ma et al. (2005) described FISH in suspension on chromosomes of barley and rye, but again without confirming suitability for flow cytometric sorting.

FISH requires changing solutions, and washing and pelleting chromosomes cause chromosome clumping and losses. Consequently, Macas et al. (1995) developed a protocol for primed in situ labeling in suspension, with a reduced number of washing and centrifugation steps. Pich et al. (1995) used the procedure to discriminate and sort field bean chromosomes based on *Fok*I repeat copy number. Unfortunately, the protocol suffered from poor reproducibility (unpublished observation). A successful attempt to label animal and human chromosomes in suspension was reported by Brind’Amour and Lansdorp (2011) who used peptide nucleic acid (PNA) probes. These probes have higher binding affinity to DNA as compared to DNA or RNA and are suitable for quantitative FISH. In this work, PNA probe to human chromosome 18-specific pericentromeric satellite facilitated the flow sorting of parental chromosome homologs, which differed in the number of repeat units. An attractive alternative to FISH is chromosome labeling using synthetic polyamide probes, which bind in a sequence-specific manner but do not require denaturation of DNA. The feasibility of this approach for labeling chromosomes in suspension was confirmed by Gygi et al. (2002) who used a polyamide targeted to a sequence motif repeated in heterochromatic regions to discriminate by flow cytometry human chromosome 9 from chromosomes 10, 11, and 12. Surprisingly, this method has not been used by others.

So far the most productive approach to sort otherwise non-sortable chromosomes has been a careful selection of genotypes from which particular chromosomes can be purified. Chromosomes that could not be sorted from samples prepared from human cells were sorted from human–hamster hybrid cell lines containing one or a few human chromosomes of interest (Lee et al. 1994; Gingrich et al. 1996). A similar approach was used to sort some pig chromosomes from pig–mouse somatic cell hybrid lines (Bouvet et al. 1993). An alternative was to use cell lines containing chromosomes with distinctive heteromorphisms, and Harris et al. (1985) demonstrated that selection of suitable lines facilitated sorting of all human chromosomes apart from 10, 11, and 12. The recent progress in plant flow cytogenetics has been stimulated mainly by the use of cytogenetic stocks. Lucretti et al. (1993) and Doležel and Lucretti (1995) showed that field bean chromosomes whose length has been altered by translocation could be easily discriminated. Since then, a whole range of cytogenetic stocks, including deletions (Fig. 4b; Gill et al. 1999; Kubaláková et al. 2002, 2005), translocations (Fig. 4f; Kubaláková et al. 2002; Neumann et al. 1998, 2002), alien chromosome addition (Fig. 4c; Kubaláková et al. 2003; Li et al. 2001), and alien chromosome arm additions (Fig. 4d; Suchánková et al. 2006), has been found useful to discriminate specific chromosomes and chromosome arms in a variety of plant species.

## The many important uses of flow-sorted chromosomes

Flow cytogenetics has become a powerful tool due to the ability to isolate particular chromosomes in purities and quantities needed for a broad range of applications. The availability of purified fractions of chromosomes greatly simplifies the analysis of complex genomes and enables experimental approaches and studies which otherwise would not be realistic. Flow cytogenetics was instrumental during the early phases of the human genome sequencing project, catalyzed the recent progress in clinical cytogenetics, contributed significantly to the analysis of karyotype evolution in primates and other animals, and assisted in physical mapping and sequencing plant genomes, to name just a few key contributions. Genome analysis using chromosome-based approaches has been termed chromosome genomics. The applications are numerous and keep on expanding along with the advances in methods of cell and molecular biology and genomics. What follows is a brief outline of major uses and applications.

### Physical mapping using DNA hybridization and PCR

Assignment of genes to particular chromosomes and subchromosomal regions was one of the first uses of sorted chromosomes. Initially, DNA was isolated from purified chromosomes and used for Southern blotting with labeled DNA probes (Lebo 1982). Later, chromosome dot-blots were prepared from only 10^4^ chromosomes sorted onto a nitro-cellulose filter disk and the chromosomal DNA was hybridized with labeled DNA probe (Lebo et al. 1984; Arumuganathan et al. 1994). This approach was replaced by PCR with specific primers, reducing the number of chromosomes needed to less than 500 (Cotter et al. 1989). PCR with sorted chromosomes has been used extensively in human, animals, and plants to localize DNA sequences to particular chromosomes (Kejnovský et al. 2001), integrate genetic and physical maps (Sargan et al. 2000; Neumann et al. 2002; Vláčilová et al. 2002), and determine breakpoints of chromosome deletions (Silverman et al. 1995) and translocations (Kamnasaran et al. 2001). Sorting both derivative chromosomes from translocation lines with balanced translocations facilitates subchromosomal mapping (Carter 1993; Macas et al. 1993). Amplification of chromosomal DNA using high-fidelity DNA polymerases (Hui et al. 1995; Šimková et al. 2008) has been used to produce DNA in microgram quantities and sufficient for many PCR reactions, thus obviating a need to sort many individual samples. It is important that the amplification is highly representative (Šimková et al. 2008).

### Physical mapping using FISH

FISH has been an important tool in physical genome mapping, for example, to anchor genetic linkage groups to particular chromosomes, establish order and orientation of contigs during the construction of physical map, and estimate the size of contig gaps (Szinay et al. 2010; Han et al. 2011). FISH has traditionally been done on mitotic metaphase spreads. Chromosomes sorted onto microscopic slides are an attractive alternative as they are completely free of cytoplasmic contamination and facilitate high-resolution analysis on large populations of chromosomes (Lucretti et al. 1993). This enabled the analysis of the intravarietal polymorphism in genomic distribution of GAA clusters in wheat (Kubaláková et al. 2002) and the identification of a rare translocation between A and B chromosomes in rye (Kubaláková et al. 2003). A further advantage of using flow-sorted chromosomes for FISH is a possibility to stretch them longitudinally up to a hundredfold compared with untreated chromosomes, making them suitable for high-resolution mapping (Valárik et al. 2004). This approach is especially attractive for plant species with large genomes as an alternative to FISH on pachytene chromosomes, which are difficult to trace individually (de Jong et al. 1999).

### Small-insert DNA libraries

Flow cytogenetics played a key role in the early stages of the human genome project in constructing chromosome-specific libraries. The first small-insert DNA library was constructed by Davies et al. (1981) from human chromosome X. In a similar work, Krumlauf et al. (1982) created libraries from autosomes 21 and 22, and ultimately two complete sets of small-insert DNA libraries for each of the 24 human chromosome types were created by the US National Laboratory Gene Library Project (Van Dilla et al. 1986; van Dilla and Deaven 1990). Comparable libraries were constructed for various animals (Baron et al. 1990; Shepel et al. 1998) and in wheat (Wang et al. 1992). Construction of short-insert libraries became easier after the introduction of methods for representative amplification of chromosomal DNA as only a few hundred or thousand sorted chromosomes (Miller et al. 1992; Vooijs et al. 1993; Macas et al. 1996) or even a single chromosome (Van Devanter et al. 1994) was sufficient as starting material. Chromosome specifics of the libraries facilitated gene mapping and targeted the development of DNA markers in human, animals, and plants (Arumuganathan et al. 1994; Grady et al. 1996; Lan et al. 1999; Korstanje et al. 2001; Požárková et al. 2002).

### Large-insert DNA libraries

Construction of physical maps and positional gene cloning requires large-insert DNA libraries. Although their construction requires large amounts of high molecular weight DNA, numerous libraries were constructed successfully from partially digested chromosomal DNA by cloning into cosmid (Stallings et al. 1990; Nizetic et al. 1994; Ma et al. 1996), fosmid (Kim et al. 1995; Gingrich et al. 1996), yeast artificial chromosome (YAC) (McCormick et al. 1993a, b), and, later, bacterial artificial chromosome (BAC) (Šafář et al. 2004; Janda et al. 2006) vectors. As the sorting of millions of chromosomes needed to construct libraries cloned in YAC and BAC vectors is a daunting task, an alternative approach has been used and genomic YAC or BAC library is constructed and screened with a probe prepared either from a chromosome-specific cosmid library (Kim et al. 1994) or from DNA from flow-sorted chromosomes (Sankovic et al. 2006) to identify clones coming from the chromosome of interest and assemble a chromosome-specific sub-library. This approach, however, is only feasible if repetitive DNA in the probe can be blocked to avoid non-specific hybridization and is not useful for plants, which are characterized by dispersed repeats (Schubert et al. 2001). In order to construct BAC libraries from DNA of sorted plant chromosomes, Šafář et al. (2004) developed a protocol which requires only a few micrograms of DNA. This advance facilitated the construction of a number of chromosome-specific BAC libraries in wheat and rye (Šafář et al. 2010). The libraries have been instrumental to establishing physical maps after restriction fragment analysis (fingerprinting) and assembling BAC contigs (Paux et al. 2008; International Wheat Genome Sequencing Consortium, http://www.wheatgenome.org/) and have been a key breakthrough in genome sequencing projects. The flow-sorted chromosome-based analysis of the wheat and barley genomes has simplified positional gene cloning especially in wheat because it is a polyploid genome, almost four times larger than that of human.

### Physical mapping and nanofluidics

Rapid development of microfluidic technology provided new opportunities for physical mapping eukaryotic genomes. One of them is optical mapping, in which high-resolution restriction maps are prepared from very long DNA molecules deposited on a slide. The maps derived from single DNA molecules are combined to produce a consensus, genomic map. Optical mapping has been shown to be particularly useful in highly repetitive and duplicated genomes to assemble their sequences and verify finished sequence data (Zhou et al. 2009; Young et al. 2011), study genome structural polymorphism (Teague et al. 2010), and perform genome-wide DNA methylation mapping (Ananiev et al. 2008). A modified approach to construct optical maps employs nanofluidic devices with a series of parallel microchannels through which DNA molecules move and can be analyzed (Das et al. 2010; Neely et al. 2011). The analysis of DNA in solution is facilitated by using nicking enzymes and fluorescent labeling of displaced single strands. The use of chromosomal DNA could greatly simplify the assembly of optical maps in organisms with large and polyploid genomes such as bread wheat, and preliminary results confirmed that DNA from flow-sorted chromosomes is suitable for optical mapping (unpublished observation).

### Development of DNA markers

A typical procedure for marker development employs genomic DNA. If, however, there is a need to develop markers from a particular genome region, this strategy is highly inefficient. A targeted alternative has been the development of markers from short-insert chromosome-specific DNA libraries (Arumuganathan et al. 1994; Grady et al. 1996; Lan et al. 1999), in some cases enriched for DNA motives of interest (Korstanje et al. 2001; Požárková et al. 2002; Kofler et al. 2008). DNA markers were also developed from clones from chromosome-specific DNA libraries with large inserts after sequencing their ends (Paux et al. 2006; Bartoš et al. 2008). Development of some types of marker such as the Diversity Array Technology markers (Jaccoud et al. 2001) does not require a prior construction of DNA libraries, and the markers can be developed directly from only a few nanograms of chromosomal DNA (Wenzl et al. 2010). A powerful approach for targeted development of markers became available thanks to the progress in mass parallel sequencing technology (Mardis 2008). Next-generation sequencing chromosomal DNA identifies enough sequences from genes and intergenic regions to develop literally an unlimited number of markers, including single nucleotide polymorphisms (SNPs) (Mayer et al. 2009, 2011; Berkman et al. 2011; Wicker et al. 2011; Fluch et al. 2012).

### Chromosome painting

Fluorescently labeled DNA from human chromosome-specific DNA libraries can be used for FISH to label specifically chromosomes in metaphase and interphase (Cremer et al. 1988; Pinkel et al. 1998). This procedure, termed chromosome painting, developed into a major tool in clinical and research molecular cytogenetics (Langer et al. 2004). Its spread was supported by protocols for amplification of chromosomal DNA (Chang et al. 1992; Telenius et al. 1992), which allowed the generation of painting probes from a small number of sorted chromosomes. Gribble et al. (2004) reported on generating chromosome paints from single copies of chromosomes. Although representative amplification of DNA from a single chromosome is demanding, this approach avoids the risk of contamination by other chromosomes and allows generating paints from chromosomes, which cannot be discriminated from other chromosomes.

**Fig. 5:**
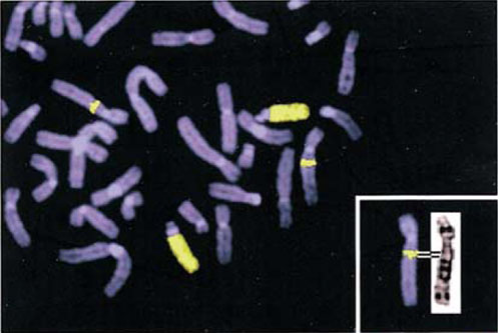
Reverse chromosome painting, using the flow-sorted DOP–PCR-amplified aberrant human chromosome 13 as a probe, defines the exact origin and breakpoints of the insertion as 5q12 to 5q13.3 (Blennow (2004), with permission)

Amplification of chromosomal DNA enabled reverse chromosome painting, in which the paint is developed from a flow-sorted aberrant chromosome of interest and hybridized to a normal karyotype to reveal the composition of the aberrant chromosome and position of chromosomal breakpoints (Fig. 5; Carter et al. 1992; Blennow 2004). Labeling the painting probes with several fluorochromes in a combinatorial approach allows identification of all 24 human chromosome types in a single experiment (Speicher et al. 1996; Schrock et al. 1996). Chromosome painting probes can be prepared also in animals; in addition, to study chromosome aberrations (Rubeš et al. 2009), major applications have included cross-species (comparative) chromosome painting, which is termed ZOO–FISH (Scherthan et al. 1994). This is a very useful technique to analyze evolution and phylogeny (Ferguson-Smith 1997; Ferguson-Smith and Trifonov 2007; Nie et al. 2012). Unfortunately, chromosome painting does not work in plants due to significant amounts of dispersed repeats in their genomes (Schubert et al. 2001). In species with small genomes and less repetitive DNA, the alternative has been to use FISH with pools of selected BAC clones (Lysák et al. 2001).

### Physical mapping on DNA arrays and array painting

Coupling DNA array technology with flow cytogenetics resulted in the so-called array painting, which allows high-resolution analysis of the content and breakpoint of aberrant chromosomes (Fiegler et al. 2003; Veltman et al. 2003). Here the painting probes are prepared from two derivative chromosomes, each of them is labeled with a different fluorochrome and both are hybridized to DNA microarray with mapped DNA sequences. Plotting the fluorescence ratio against the clone position along each chromosome provides information on chromosome composition (Le Scouarnec and Gribble 2012). Originally, the DNA sequences were DNA clones, and arrays with 30,000 BAC clones, which became available in human, providing 100 kb resolution (Curtis et al. 2009). If the sequence of a clone spanned chromosome breakpoint, the exact position of the breakpoint could be determined. Further improvement of the technology led to an increased number of features on the array and use of shorter sequences as targets—most frequently oligonucleotides. Thus, Conrad et al. (2010) used a set of 20 ultra-high resolution oligonucleotide arrays comprising 42 million probes in total, with a median probe spacing of just 56 bp across the entire human genome. Similar arrays are becoming commercially available for some animals.

DNA arrays are becoming available also for non-sequenced plants and recent results obtained in barley confirmed the great potential of DNA arrays used with sorted chromosomes for physical mapping. Šimková et al. (2008) mapped 162 SNP loci, including 40 loci with hitherto unknown map position to barley chromosome 1H using a pilot oligonucleotide pool assay. In a larger-scale study, Mayer et al. (2011) used DNA from flow-sorted barley chromosome 1H and arms of chromosomes 2H–7H on barley 44k Agilent microarray to assign 16,804 genes to individual chromosomes. During a development of a consensus genetic map of barley, the authors used two barley oligonucleotide pool assays to examine 3,072 SNP markers with DNA from sorted barley chromosome 1H and arms of chromosomes 2H–7H. As chromosome location is independent of the allele, the mapping was robust and the authors mapped 2,930 genes (96.1 % of total genes surveyed). An additional 370 genes were mapped using flow-sorted materials, which were not genetically mapped in any of the ten mapping populations used. Finally, when coupled with the consensus genetic map, gene mapping using flow-sorted chromosome arms permitted the definition of pericentromeric regions in chromosomes 2H–7H (Muñoz-Amatriaín et al. 2011).

### Chromosome sequencing using next-generation technology

**Fig. 6:**
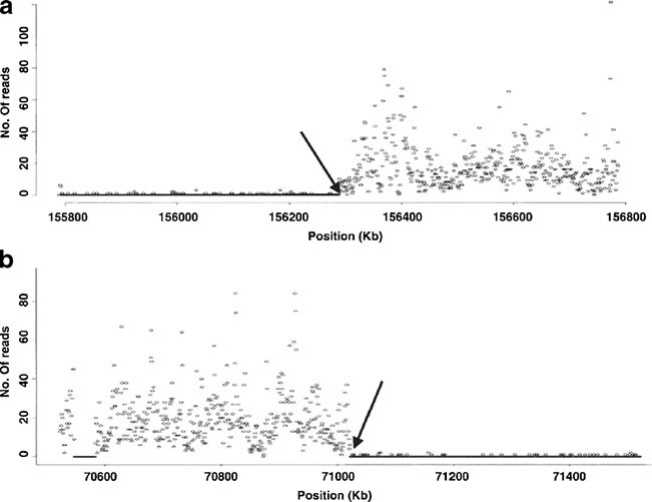
Solexa sequencing profile of human derivative chromosome 9. Shown are 1-Mb intervals around the breakpoints (*arrow*s) on chromosome 7 (**a**) and 9 (**b**). A total of 199,421 and 1,047,649 reads derived from the derivative chromosome 9 were mapped to unique positions on normal chromosomes 7 and 9, respectively. The number of reads was then binned into non-overlapping 1-kb segments and plotted against the chromosome coordinates (Chen et al. (2008), with permission)

In species with sequenced genomes, re-sequencing chromosomes is a rapid means for studying variation at DNA level by aligning short reads to the reference sequence. Sequencing single chromosomes reduces costs and simplifies data analysis as compared to whole genomes. As demonstrated by Chen et al. (2008, 2010), massively parallel sequencing of flow-sorted derivative chromosomes is an elegant approach to determine the chromosome composition and map chromosomal breakpoints with an error margin of less than 1,000 bp (Fig. 6). With the falling sequencing costs, this approach is expected to replace array painting. In mouse, Sudbery et al. (2009) confirmed that whole-chromosome sequencing allows generating dense maps of genetic variation between different genotypes and that it is a powerful approach for SNP discovery, deriving a high-resolution picture of QTL regions.

**Fig. 7:**
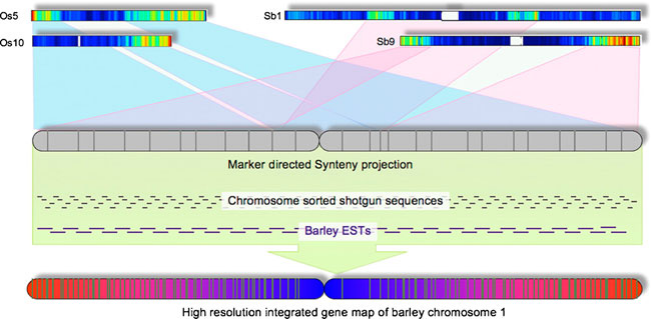
Schematic representation of marker- and synteny-guided assembly of an integrated virtual gene map for barley chromosome 1 H. Genetically anchored barley markers have been integrated with rice and sorghum genes located in syntenic regions to give an enriched tentative ancestral gene scaffold. Sequence reads from flow-sorted barley chromosome 1H as well as barley EST sequences have been associated with this chromosome matrix and give rise to an ordered integrated gene map of the chromosome (Mayer et al. (2009), with permission)

Massively parallel sequencing of chromosomal DNA is perhaps even more attractive in organisms for which genome sequence is not available. Mayer et al. (2009) demonstrated that low-pass 454 sequencing flow-sorted barley chromosome 1H (1.3-fold coverage) was a cost-effective approach to describe gene content, assess gene synteny with other species, and establish comprehensive linear gene-order model for the chromosome (Fig. 7). This work was expanded to wheat by Wicker et al. (2011) who studied the molecular structure and gene content of homoeologous chromosome group 1 of hexaploid wheat. Low-pass 454 sequencing of all chromosome of barley (2.2-fold average coverage) by Mayer et al. (2011) resulted in a blueprint of the barley genome reaching—at a fraction of the costs—a level of information density and resolution, which can be surpassed only by whole genome sequencing. Sequencing wheat chromosome 5A by 454 revealed the main sequence features of this chromosome, including candidate miRNA precursors, and enabled the production of a virtual gene order based on synteny with other phylogenetically related species (Vitulo et al. 2011). The same method was used to sequence wheat chromosome 4A. Hernandez et al. (2012) built an ordered gene map of chromosome 4A and localized precisely translocations from chromosomes 5A and 7B and inversion breakpoints on this most rearranged chromosome of wheat. Fluch et al. (2012) sequenced by 454 the short arm of rye chromosome 1R, which is present in many cultivars of bread wheat in the form of translocation chromosome 1RS.1BL. Among others, this work permitted a detailed description of the gene space as well as the repetitive portion of the chromosome.

In a similar work, Berkman et al. (2011) characterized the short arm of wheat chromosome 7D (7DS) after sequencing by Illumina to 34-fold coverage. Despite relatively short reads (paired reads of 75, 76, or 100 bp and an insert size of 320 bp), they succeeded in assembling approximately 40 % of 7DS and all known genes. They used syntenic relationship between wheat and a sequenced close relative *Brachypodium distachyon* to produce annotated syntenic builds whereby the majority of genes have been placed in an approximate order and orientation. Subsequently, Berkman et al. (2012) characterized wheat chromosome arm 7BS in the same way. In addition to describing gene content, they delimited the position of a previously described translocation between 7BS and 4AL with a resolution of one or a few genes and reported approximately 13 % genes from 7BS to have been translocated to 4AL. An additional 13 genes were found on 7BS, which appeared to originate from 4AL. With the unprecedented gene density and resolution obtained at a fraction of cost of full-scale sequencing, next-generation sequencing of flow-sorted chromosomes is bound to aid greatly in gene mapping and cloning and the analysis of genome evolution. Heat maps used to graphically depict positions of chromosome sequence reads in genomes of related species resemble the classical comparative painting. However, electronic chromosome painting (E-painting; Kemkemer et al. 2006) results in much higher resolution and may be performed also in plants with repeat-rich genomes.

Flow cytometric sorting is attractive because of its capacity to purify large numbers of chromosomes. However, flow sorters can also be used to sort single copies of chromosomes. Yang et al. (2011) took advantage of this and sequenced DNA amplified from single copies of chromosome 19 and demonstrated the utility of this approach, called Phase-Seq, to analyze phase information between parental allelic sequences. If this result is confirmed, flow cytogenetics may play an important role in producing haplotype-resolved genome sequences. In fact, sequencing DNA from single chromosome copies may be a solution in those cases where it is not possible to discriminate single chromosome types. Sequencing pools of DNA amplified from single copies of the same chromosome may provide sufficient sequence coverage of any chromosome of interest. This application of flow cytogenetics may be an elegant alternative to the recently developed microfluidic approach, in which individual chromosomes from a single human metaphase are separated into distinct channels and amplified (Fan et al. 2011).

### Higher-order structure and proteins of mitotic chromosomes

In a majority of research, flow cytogenetics has been employed to aid in analyzing chromosomal DNA. However, there are as yet not fully explored opportunities to analyze the higher-order structure of mitotic chromosomes and their major component—the chromosomal proteins. Trask et al. (1984) demonstrated the ability to label immunofluorescently histones and centromeric proteins on mitotic chromosomes and classify the fluorescence by flow cytometry. Unfortunately, the differences in immunofluorescent staining of centromeric proteins between chromosomes were only minor and the labeling did not allow identifying dicentric chromosomes to quantify the effect of radiation, most probably due to non-specific antibody binding (Fantes et al. 1989). Schubert et al. (1993) demonstrated that isolated plant chromosomes were suitable for immunostaining of chromosomal antigens and this property enabled a detailed analysis of plant kinetochore proteins (Binarová et al. 1998; ten Hoopen et al. 2000).

Recent progress in proteomics offers a possibility to describe all proteins of mitotic chromosomes. A pioneering work of Uchiyama et al. (2005) led to the identification of 107 proteins in human chromosomes and a proposal of a four-layer model of metaphase chromosomes (Takata et al. 2007; Fukui 2009). In these studies, proteins were isolated from chromosomes purified on a sucrose gradient. However, incorporating the isolation of chromosomes by flow sorting as part of protein analyses might result in higher sample purity, and this option is worth exploring. This work may contribute significantly towards characterizing the protein component of mitotic chromosomes and understanding the determination of centromere, a process which seems to depend primarily on protein component and its posttranslational modification (Birchler et al. 2009).

The suitability of isolated chromosomes for scanning electron microscopy to study their higher-order structure was demonstrated already by Schubert et al. (1993). Compared to other protocols (Wanner et al. 1991), large numbers of chromosomes may be prepared and the preparations are not covered by remnants of cytoplasm, which obscure surface details. However, the opportunity to use flow-sorted mitotic chromosomes to study their higher-order structure has not been addressed so far.

### Development of artificial chromosomes

Artificial chromosomes or engineered mini-chromosomes are safe and stable non-integrating vectors developed to carry large segments of genomic DNA. They hold a great promise for gene therapy, animal biotechnology, and plant breeding (Duncan and Hadlaczky 2007; Birchler et al. 2010). They have been developed either via bottom-up approach using cloned components of chromosomes or top-down approach through the truncation of existing chromosomes (Goyal et al. 2009; Hoshiya et al. 2009). Mini-chromosomes derived by the top-down approach are more natural systems for maintaining and expressing transgenes (Macnab and Whitehouse 2009; Birchler et al. 2010). One of the limiting factors hampering routine therapeutic and other applications is the purification of high quantities of artificial chromosomes, and flow cytometry has been considered suitable for this task (Lindenbaum et al. 2004). The advances in parallel flow sorting technology make it possible to sort up to one billion particles per hour (Wlodkowic and Darzynkiewicz 2011). The minimum size of a chromosome to segregate to a high fidelity during mitotic division in human is 10 Mb (Macnab and Whitehouse 2009), and Ng et al. (2007) demonstrated the ability to distinguish and flow-sort chromosomes to be smaller than 3 Mbp; thus, flow cytometry offers the required sensitivity and throughput.

## Conclusions

Flow cytometric chromosome analysis and sorting (flow cytogenetics) is a unique technology which requires specialized equipment and thus has never been mastered by many research teams. This contrasts with the enormous impact the technology has made during the past 35 years in many areas of basic and applied research in human and many animal and plant species. Success was possible also due to the fact that molecular chromosome resources could be distributed worldwide from a few specialized laboratories. This mode of work stimulates international collaborations in which several laboratories work in parallel on different chromosomes. For example, the availability of chromosome-specific DNA libraries greatly facilitated the initial phases of the human genome sequencing project. In wheat, the production and distribution of chromosome arm-specific BAC libraries has underpinned the current international effort to sequence the huge genome (Feuillet and Eversole 2007; International Wheat Genome Sequencing Consortium, http://www.wheatgenome.org/). Production of chromosome painting probes revolutionized clinical and research cytogenetics and provided an instrument to study structural chromosome changes accompanying genome evolution in human and many lineages of animals. Dissecting large and complex genomes of some crops to smaller, chromosome-based parts has facilitated the construction of physical maps, positional gene cloning, and genome sequencing. Coupling flow cytogenetics with high-resolution DNA arrays and mass parallel sequencing has led to new applications with enormous potential for genome analysis and suggests that technology will continue to play a significant role in many areas of genetic and genomic research.

## References

[CR1] lkan C, Sajjadian S, Eichler EE (2011). Limitations of next-generation genome sequence assembly. Nat Methods.

[CR2] Ananiev GE, Goldstein S, Runnheim R, Forrest DK, Zhou SG, Potamousis K, Churas CP, Bergendahl V, Thomson JA, Schwartz DC (2008). Optical mapping discerns genome wide DNA methylation profiles. BMC Mol Biol.

[CR3] Arumuganathan K, Slattery JP, Tanksley SD, Earle ED (1991). Preparation and flow cytometric analysis of metaphase chromosomes of tomato. Theor Appl Genet.

[CR4] Arumuganathan K, Martin GB, Telenius H, Tanksley SD, Earle ED (1994). Chromosome 2-specific DNA clones from flow-sorted chromosomes of tomato. Mol Gen Genet.

[CR5] Aten JA, Buys CHCM, Vanderveen AY, Mesa JR, Yu LC, Gray JW, Osinga J, Stap J (1987). Stabilization of chromosomes by DNA intercalators for flow karyotyping and identification by banding of isolated chromosomes. Histochemistry.

[CR6] Aten JA, Kooi MW, Stap J, Kipp JBA, Barendsen GW (1987). X-ray-induced and neutron-induced chromosome-damage detected by flow-cytometry compared to cell lethality and chromosome structural-changes. Radiat Res.

[CR7] Barigozzi C (1939). Experiments with microdissection of the chromosome of the salivary glands of *Chironomus* sp. Archiv Exp Zellforsch.

[CR8] Baron B, Metezeau P, Kiefergachelin H, Goldberg ME (1990). Construction and characterization of a DNA library from mouse chromosomes-19 purified by flow-cytometry. Biol Cell.

[CR9] Bartholdi MF (1990). Flow cytogenetics. Pathobiology.

[CR10] Bartholdi MF, Meyne J, Johnston RG, Cram LS (1989). Chromosome-banding analysis by slit-scan flow-cytometry. Cytometry.

[CR11] Bartholdi MF, Parson JD, Albright KA, Cram LS (1990). System for flow sorting chromosomes on the basis of pulse shape. Cytometry.

[CR12] Bartoš J, Paux E, Kofler R, Havránková M, Kopecký D, Suchánková P, Šafář J, Šimková H, Town CD, Lelley T, Feuillet C, Doležel J (2008). A first survey of the rye (*Secale cereale*) genome composition through BAC end sequencing of the short arm of chromosome 1R. BMC Plant Biol.

[CR13] Berkman PJ, Skarshewski A, Lorenc MT, Lai K, Duran C, Ling EY, Stiller J, Smits L, Imelfort M, Manoli S, McKenzie M, Kubaláková M, Šimková H, Batley J, Fleury D, Doležel J, Edwards D (2011). Sequencing and assembly of low copy and genic regions of isolated *Triticum aestivum* chromosome arm 7DS. Plant Biotechnol J.

[CR14] Berkman PJ, Skarshewski A, Manoli S, Lorenc MT, Stiller J, Smits L, Lai K, Campbell E, Kubaláková M, Šimková H, Batley J, Doležel J, Hernandez P, Edwards D (2012). Sequencing wheat chromosome arm 7BS delimits the 7BS/4AL translocation and reveals homoeologous gene conservation. Theor Appl Genet.

[CR15] Bijman JT (1983). Optimization of mammalian chromosome suspension preparations employed in a flow cytometric analysis. Cytometry.

[CR16] Binarová P, Hause B, Doležel J, Dráber P (1998). Association of γ-tubulin with kinetochore/centromeric region of plant chromosomes. Plant J.

[CR17] Birchler J, Gao Z, Han FP (2009). A tale of two centromeres—diversity of structure but conservation of function in plants and animals. Funct Integr Genom.

[CR18] Birchler JA, Krishnaswamy L, Gaeta RT, Masonbrink RE, Zhao CZ (2010). Engineered minichromosomes in plants. Crit Rev Plant Sci.

[CR19] Blennow E (2004). Reverse painting highlights the origin of chromosome aberrations. Chromosom Res.

[CR20] Boschman GA, Manders EMM, Rens W, Slater R, Aten JA (1992). Semiautomated detection of aberrant chromosomes in bivariate flow karyotypes. Cytometry.

[CR21] Bouvet A, Konfortov BA, Miller NGA, Brown D, Tucker EM (1993). Identification of pig chromosomes in pig–mouse somatic-cell hybrid bivariate flow karyotypes. Cytometry.

[CR22] Brar GA, Amon A (2008). Emerging roles for centromeres in meiosis I chromosome segregation. Nat Rev Genet.

[CR23] Brind’Amour J, Lansdorp PM (2011). Analysis of repetitive DNA in chromosomes by flow cytometry. Nat Methods.

[CR24] Carrano AV, Gray JW, Moore DH, Minkler JL, Mayall BH, Van Dilla MA, Mendelsohn ML (1976). Purification of the chromosomes of the Indian muntjac by flow sorting. J Histochem Cytochem.

[CR25] Carrano AV, Gray JW, Langlois RG, Burkhartschultz KJ, Van Dilla MA (1979). Measurement and purification of human-chromosomes by flow cytometry and sorting. Proc Natl Acad Sci USA.

[CR26] Carter NP, Jacquemin-Sablon A (1993). Gene mapping and PCR application with flow-sorted chromosomes. Flow cytometry.

[CR27] Carter NP, Ferguson-Smith MA, Perryman MT, Telenius H, Pelmear AH, Leversha MA, Glancy MT, Wood SL, Cook K, Dyson HM (1992). Reverse chromosome painting: a method for the rapid analysis of aberrant chromosomes in clinical cytogenetics. J Med Genet.

[CR28] Chambers R, Sands C (1923). A dissection of the chromosomes in the pollen mother cells of *Tradescantia virginiana*. J Gen Physiol.

[CR29] Chang KS, Vyas RC, Deaven LL, Trujillo JM, Stass SA, Hittelman WN (1992). PCR amplification of chromosome-specific DNA isolated from flow cytometry-sorted chromosomes. Genomics.

[CR30] Chen W, Kalscheuer V, Tzschach A, Menzel C, Ullmann R, Schulz MH, Erdogan F, Li N, Kijas Z, Arkesteijn G, Pajares IL, Goetz-Sothmann M, Heinrich U, Rost I, Dufke A, Grasshoff U, Glaeser B, Vingron M, Ropers HH (2008). Mapping translocation breakpoints by next-generation sequencing. Genome Res.

[CR31] Chen W, Ullmann R, Langnick C, Menzel C, Wotschofsky Z, Hu H, Döring A, Hu Y, Kang H, Tzschach A, Hoeltzenbein M, Neitzel H, Markus S, Wiedersberg E, Kistner G, van Ravenswaaij-Arts CM, Kleefstra T, Kalscheuer VM, Ropers HH (2010). Breakpoint analysis of balanced chromosome rearrangements by next-generation paired-end sequencing. Eur J Hum Genet.

[CR32] Conia J, Bergounioux C, Perennes C, Muller P, Brown S, Gadal P (1987). Flow cytometric analysis and sorting of plant chromosomes from *Petunia hybrida* protoplasts. Cytometry.

[CR33] Conia J, Muller P, Brown S, Bergounioux C, Gadal P (1989). Monoparametric models of flow cytometric karyotypes with spreadsheet software. Theor Appl Genet.

[CR34] Conrad DF, Pinto D, Redon R, Feuk L, Gokcumen O, Zhang Y, Aerts J, Andrews TD, Barnes C, Campbell P, Fitzgerald T, Hu M, Ihm CH, Kristiansson K, Macarthur DG, Macdonald JR, Onyiah I, Pang AW, Robson S, Stirrups K, Valsesia A, Walter K, Wei J, Wellcome Trust Case Control C, Tyler-Smith C, Carter NP, Lee C, Scherer SW, Hurles ME (2010). Origins and functional impact of copy number variation in the human genome. Nature.

[CR35] Cotter F, Nasipuri S, Lam G, Young BD (1989). Gene mapping by enzymatic amplification from flow-sorted chromosomes. Genomics.

[CR36] Cram LS, Bartholdi MF, Ray FA, Travis GL, Kraemer PM (1983). Spontaneous neoplastic evolution of Chinese-hamster cells in culture—multistep progression of karyotype. Cancer Res.

[CR37] Cram LS, Bell CS, Fawcett JJ (2002). Chromosome sorting and genomics. Methods Cell Sci.

[CR38] Cremer T, Lichter P, Borden J, Ward DC, Manuelidis L (1988). Detection of chromosome aberrations in metaphase and interphase tumor cells by in situ hybridization using chromosome-specific library probes. Hum Genet.

[CR39] Crosland MWJ, Crozier RH (1986). *Myrmecia pilosula*, an ant with only one pair of chromosomes. Science.

[CR40] Curtis C, Lynch AG, Dunning MJ, Spiteri I, Marioni JC, Hadfield J, Chin SF, Brenton JD, Tavare S, Caldas C (2009). The pitfalls of platform comparison: DNA copy number array technologies assessed. BMC Genomics.

[CR41] Das SK, Austin MD, Akana MC, Deshpande P, Cao H, Xiao M (2010). Single molecule linear analysis of DNA in nano-channel labeled with sequence specific fluorescent probes. Nucleic Acids Res.

[CR42] Davies KE, Young BD, Elles RG, Hill ME, Williamson R (1981). Cloning of a representative genomic library of the human X chromosome after sorting by flow cytometry. Nature.

[CR43] de Jong JH, Fransz P, Zabel P (1999). High resolution FISH in plants—techniques and applications. Trends Plant Sci.

[CR44] de Laat AMM, Blaas J (1984). Flow-cytometric characterization and sorting of plant chromosomes. Theor Appl Genet.

[CR45] de Laat AMM, Schel JHN (1986). The integrity of metaphase chromosomes of *Haplopappus gracilis* (Nutt) Gray isolated by flow cytometry. Plant Sci.

[CR46] Di Bucchianico S, Poma AM, Giardi MF, Di Leandro L, Valle F, Biscarini F, Botti D (2011). Atomic force microscope nanolithography on chromosomes to generate single-cell genetic probes. J Nanobiotechnol.

[CR47] Doležel J, Lucretti S (1995). High-resolution flow karyotyping and chromosome sorting in *Vicia faba* lines with standard and reconstructed karyotypes. Theor Appl Genet.

[CR48] Doležel J, Číhalíková J, Lucretti S (1992). A high-yield procedure for isolation of metaphase chromosomes from root tips of *Vicia faba* L. Planta.

[CR49] Doležel J, Lucretti S, Schubert I (1994). Plant chromosome analysis and sorting by flow cytometry. Crit Rev Plant Sci.

[CR50] Doležel J, Kubaláková M, Bartoš J, Macas J (2004). Flow cytogenetics and plant genome mapping. Chromosom Res.

[CR51] Dudin G, Cremer T, Schardin M, Hausmann M, Bier F, Cremer C (1987). A method for nucleic acid hybridization to isolated chromosomes in suspension. Hum Genet.

[CR52] Dudin G, Steegmayer EW, Vogt P, Schnitzer H, Diaz E, Howell KE, Cremer T, Cremer C (1988). Sorting of chromosomes by magnetic separation. Hum Genet.

[CR53] Duncan A, Hadlaczky G (2007). Chromosomal engineering. Curr Opin Biotechnol.

[CR54] Fan HC, Wang JB, Potanina A, Quake SR (2011). Whole-genome molecular haplotyping of single cells. Nat Biotechnol.

[CR55] Fantes JA, Green DK, Elder JK, Malloy P, Evans HJ (1983). Detecting radiation damage to human chromosomes by flow cytometry. Mutat Res.

[CR56] Fantes JA, Green DK, Malloy P, Sumner AT (1989). Flow-cytometry measurements of human-chromosome kinetochore labeling. Cytometry.

[CR57] Ferguson-Smith MA (1997). Genetic analysis by chromosome sorting and painting: phylogenetic and diagnostic applications. Eur J Hum Genet.

[CR58] Ferguson-Smith MA, Trifonov V (2007). Mammalian karyotype evolution. Nat Rev Genet.

[CR59] Feuillet C, Eversole K (2007). Physical mapping of the wheat genome: a coordinated effort to lay the foundation for genome sequencing and develop tools for breeders. Isr J Plant Sci.

[CR60] Fiegler H, Gribble SM, Burford DC, Carr P, Prigmore E, Porter KM, Clegg S, Crolla JA, Dennis NR, Jacobs P, Carter NP (2003). Array painting: a method for the rapid analysis of aberrant chromosomes using DNA microarrays. J Med Genet.

[CR61] Fluch S, Kopecky D, Burg K, Šimková H, Taudien S, Petzold A, Kubaláková M, Platzer M, Berenyi M, Krainer S, Doležel J, Lelley T (2012). Sequence composition and gene content of the short arm of rye (*Secale cereale*) chromosome 1. PLoS One.

[CR62] Fuchs J, Houben A, Brandes A, Schubert I (1996). Chromosome ‘painting’ in plants—a feasible technique?. Chromosoma.

[CR63] Fukui K (2009). Structural analyses of chromosomes and their constituent proteins. Cytogenet Genome Res.

[CR64] Gill KS, Arumuganathan K, Le JH (1999). Isolating individual wheat (*Triticum aestivum*) chromosome arm by flow cytometric analysis of ditelosomic lines. Theor Appl Genet.

[CR65] Gingrich JC, Boehrer DM, Garnes JA, Johnson W, Wong BS, Bergmann A, Eveleth GG, Langlois RG, Carrano AV (1996). Construction and characterization of human chromosome 2-specific cosmid, fosmid, and PAC clone libraries. Genomics.

[CR66] Gordon DJ, Resio B, Pellman D (2012). Causes and consequences of aneuploidy in cancer. Nat Rev Genet.

[CR67] Goyal A, Bhowmik PK, Basu SK (2009). Minichromosomes: the second generation genetic engineering tool. Plant Omics.

[CR68] Grady DL, Robinson DL, Gersh M, Nickerson E, McPherson J, Wasmuth JJ, Overhauser J, Deaven LL, Moyzis RK (1996). The generation and regional localization of 303 new chromosome 5 sequence-tagged sites. Genomics.

[CR69] Gray JW (1989). Flow cytogenetics.

[CR70] Gray JW, Cram LS, Melamed MR, Mendelsohn ML (1990). Flow karyotyping and chromosome sorting. Flow cytometry and chromosome sorting.

[CR71] Gray JW, Carrano AV, Steinmetz LL, Van Dilla MA, Moore HH, Mayall BH, Mendelsohn ML (1975). Chromosome measurement and sorting by flow systems. Proc Natl Acad Sci USA.

[CR72] Gray JW, Carrano AV, Moore HH, Steinmetz LL, Minkler J, Mayall BH, Mendelsohn ML, Van Dilla MA (1975). High-speed quantitative karyotyping by flow microfluorometry. Clin Chem.

[CR73] Gray JW, Lucas J, Peters D, Pinkel D, Trask B, van den Engh G, Van Dilla M (1986). Flow karyotyping and sorting of human chromosomes. Cold Spring Harb Symp Quant Biol.

[CR74] Gribble SM, Ng BL, Prigmore E, Burford DC, Carter NP (2004). Chromosome paints from single copies of chromosomes. Chromosom Res.

[CR75] Gualberti G, Doležel J, Macas J, Lucretti S (1996). Preparation of pea (*Pisum sativum* L.) chromosome and nucleus suspensions from single root tips. Theor Appl Genet.

[CR76] Gygi MP, Ferguson MD, Mefford HC, Lund KP, O’Day C, Zhou P, Friedman C, van den Engh G, Stolowitz ML, Trask BJ (2002). Use of fluorescent sequence-specific polyamides to discriminate human chromosomes by microscopy and flow cytometry. Nucleic Acids Res.

[CR77] Han YH, Zhang ZH, Huang SW, Jin WW (2011). An integrated molecular cytogenetic map of *Cucumis sativus* L. chromosome 2. BMC Genet.

[CR78] Harris P, Boyd E, Ferguson-Smith MA (1985). Optimising human chromosome separation for the production of chromosome-specific DNA libraries by flow sorting. Hum Genet.

[CR79] Harris P, Morton CC, Guglielmi P, Li F, Kelly K, Latt SA (1986). Mapping by chromosome sorting of several gene probes, including c-myc, to the derivative chromosomes of a 3;8 translocation associated with familial renal cancer. Cytometry.

[CR80] Hausmann M, Popescu CP, Boscher J, Kerboeuf D, Dölle J, Cremer C (1993). Identification and cytogenetic analysis of an abnormal pig chromosome for flow cytometry and sorting. Z Naturforsch C.

[CR81] Hernandez P, Martis M, Dorado G, Pfeifer M, Gálvez S, Schaaf S, Jouve N, Šimková H, Valárik M, Doležel J, Mayer KFX (2012). Next-generation sequencing and syntenic integration of flow-sorted arms of wheat chromosome 4A exposes the chromosome structure and gene content. Plant J.

[CR82] Heslop-Harrison JS, Schwarzacher T (2011). Organisation of the plant genome in chromosomes. Plant J.

[CR83] Hobza R, Vyskot B (2007). Laser microdissection-based analysis of plant sex chromosomes. Methods Cell Biol.

[CR84] Hoshiya H, Kazuki Y, Abe S, Takiguchi M, Kajitani N, Watanabe Y, Yoshino T, Shirayoshi Y, Higaki K, Messina G, Cossu G, Oshimura M (2009). A highly stable and nonintegrated human artificial chromosome (HAC) containing the 2.4 Mb entire human dystrophin gene. Mol Ther.

[CR85] Hui SM, Trask B, Vandenengh G, Bartuski AJ, Smith A, Flint A, Lalande M, Silverman GA (1995). Analysis of randomly amplified flow-sorted chromosomes using the polymerase chain-reaction. Genomics.

[CR86] International Wheat Genome Sequencing Consortium. http://www.wheatgenome.org/. Accessed on 14 March 2012.

[CR87] Jaccoud D, Peng K, Feinstein D, Kilian A (2001). Diversity Arrays: a solid state technology for sequence information independent genotyping. Nucleic Acids Res.

[CR88] Janda J, Šafář J, Kubaláková M, Bartoš J, Kovářová P, Suchánková P, Pateyron S, Číhalíková J, Sourdille P, Šimková H, Fairaivre-Rampant P, Hřibová E, Bernard M, Lukaszewski A, Doležel J, Chalhoub B (2006). Advanced resources for plant genomics: BAC library specific for the short arm of wheat chromosome 1B. Plant J.

[CR89] Kamnasaran D, O’Brien PCM, Schuffenhauer S, Quarrell O, Lupski JR, Grammatico P, Ferguson-Smith MA, Cox DW (2001). Defining the breakpoints of proximal chromosome 14q rearrangements in nine patients using flow-sorted chromosomes. Am J Med Genet.

[CR90] Kejnovský E, Vrána J, Matsunaga S, Souček P, Široký J, Doleže J, Vyskot B (2001). Localization of male-specifically expressed *MROS* genes of *Silene latifolia* by PCR on flow-sorted sex chromosomes and autosomes. Genetics.

[CR91] Kemkemer C, Kohn M, Kehrer-Sawatzki H, Minich P, Hogel J, Froenicke L, Hameister H (2006). Reconstruction of the ancestral ferungulate karyotype by electronic chromosome painting (E-painting). Chromosom Res.

[CR92] Kim UJ, Shizuya H, Birren B, Slepak T, deJong P, Simon MI (1994). Selection of chromosome 22-specific clones from human genomic BAC library using a chromosome-specific cosmid library pool. Genomics.

[CR93] Kim UJ, Shizuya H, Sainz J, Garnes J, Pulst SM, deJong P, Simon MI (1995). Construction and utility of a human chromosome 22-specific Fosmid library. Genet Anal Biomol Eng.

[CR94] Kofler R, Bartoš J, Gong L, Stift G, Suchánková P, Šimková H, Berenyi M, Burg K, Doležel J, Lelley T (2008). Development of microsatellite markers specific for the short arm of rye (*Secale cereale* L.) chromosome 1. Theor Appl Genet.

[CR95] Kooi MW, Aten JA, Stap J, Kipp JBA, Barendsen GW (1984). Preparation of chromosome suspensions from cells of a solid experimental rumor for measurement by flow-cytometry. Cytometry.

[CR96] Kopecký D, Číhalíková J, Kopecká J, Vrána J, Havránková M, Stočes Š, Bartoš J, Šimková H, Šafář J, Doležel J (2011) Establishing chromosome genomics in forage grasses. In: Abstracts of the “Eucarpia—29th Fodder Crops and Amenity Grasses Section Meeting”, Dublin, p 24

[CR97] Korstanje R, Gillissen GF, den Bieman MG, Versteeg SA, van Oost B, Fox RR, van Lith HA, van Zutphen LFM (2001). Mapping of rabbit chromosome 1 markers generated from a microsatellite-enriched chromosome-specific library. Anim Genet.

[CR98] Kovářová P, Navrátilová A, Macas J, Doležel J (2007). Chromosome analysis and sorting in *Vicia sativa* using flow cytometry. Biol Plant.

[CR99] Krumlauf R, Jeanpierre M, Young BD (1982). Construction and characterization of genomic libraries from specific human chromosomes. Proc Natl Acad Sci USA.

[CR100] Kubaláková M, Lysák MA, Vrána J, Šimková H, Číhalíková J, Doležel J (2000). Rapid identification and determination of purity of flow-sorted plant chromosomes using C-PRINS. Cytometry.

[CR101] Kubaláková M, Vrána J, Číhalíková J, Šimková H, Doležel J (2002). Flow karyotyping and chromosome sorting in bread wheat (*Triticum aestivum* L.). Theor Appl Genet.

[CR102] Kubaláková M, Valárik M, Bartoš J, Vrána J, Číhalíková J, Molnár-Láng M, Doležel J (2003). Analysis and sorting of rye (*Secale cereale* L.) chromosomes using flow cytometry. Genome.

[CR103] Kubaláková M, Kovářová P, Suchánková P, Číhalíková J, Bartoš J, Lucretti S, Watanabe N, Kianian SF, Doležel J (2005). Chromosome sorting in tetraploid wheat and its potential for genome analysis. Genetics.

[CR104] Lan H, Shepel LA, Haag JD, Gould MN (1999). Linkage mapping of rat chromosome 5 markers generated from chromosome-specific libraries. Mamm Genome.

[CR105] Langer S, Kraus J, Jentsch I, Speicher MR (2004). Multicolor chromosome painting in diagnostic and research applications. Chromosom Res.

[CR106] Langford CF, Fischer PE, Binns MM, Holmes NG, Carter NP (1996). Chromosome-specific paints from a high-resolution flow karyotype of the dog. Chromosom Res.

[CR107] Langlois RG, Yu L-C, Gray JW, Carrano AV (1982). Quantitative karyotyping of human chromosomes by dual beam flow cytometry. Proc Natl Acad Sci USA.

[CR108] Le Scouarnec S, Gribble SM (2012). Characterising chromosome rearrangements: recent technical advances in molecular cytogenetics. Heredity.

[CR109] Lebo RV (1982). Chromosome sorting and DNA sequence localization: a review. Cytometry.

[CR110] Lebo RV, Gorin F, Fleterick RJ, Kao FT, Cheung MC, Bruce BD, Kan YW (1984). High-resolution chromosome sorting and DNA spot-blot analysis assign McArdles syndrome to chromosome-11. Science.

[CR111] Lebo RV, Golbus MS, Cheung MC (1986). Detecting abnormal human-chromosome constitutions by dual laser flow cytogenetics. Am J Med Genet.

[CR112] Lee JH, Arumuganathan K (1999). Metaphase chromosome accumulation and flow karyotypes in rice (*Oryza sativa* L.) root tip meristem cells. Mol Cells.

[CR113] Lee JY, Koi M, Stanbridge EJ, Oshimura M, Kumamoto AT, Feinberg AP (1994). Simple purification of human-chromosomes to homogeneity using muntjac hybrid-cells. Nat Genet.

[CR114] Lee JH, Arumuganathan K, Kaeppler SM, Papa CM, Kaeppler HF (1996). Cell synchronization and isolation of metaphase chromosomes from maize (*Zea mays* L.) root tips for flow cytometric analysis and sorting. Genome.

[CR115] Lee JH, Arumuganathan K, Yen Y, Kaeppler S, Kaeppler H, Baenziger PS (1997). Root tip cell cycle synchronization and metaphase-chromosome isolation suitable for flow sorting in common wheat (*Triticum aestivum* L.). Genome.

[CR116] Lee JH, Arumuganathan K, Chung YS, Kim KY, Chung WB, Bae KS, Kim DH, Chung DS, Kwon OC (2000). Flow cytometric analysis and chromosome sorting of barley (*Hordeum vulgare* L.). Mol Cells.

[CR117] Lee JH, Arumuganathan K, Kaeppler SM, Park SW, Kim KY, Chung YS, Kim DH, Fukui K (2002). Variability of chromosomal DNA contents in maize (*Zea mays* L.) inbred and hybrid lines. Planta.

[CR118] Leitch AR, Schwarzacher T, Wang ML, Leitch IJ, Surlan-Momirovich G, Moore G, Heslop-Harrison JS (1993). Molecular cytogenetic analysis of repeated sequences in a long term wheat suspension culture. Plant Cell Tissue Organ Cult.

[CR119] Levy HP, Schultz RA, Ordonez JV, Cohen MM (1991). Anti-kinetochore staining for single laser, bivariate flow sorting of Indian muntjac chromosomes. Cytometry.

[CR120] Li IJ, Arumuganathan K, Rines HW, Phillips RL, Riera-Lizarazu O, Sandhu D, Zhou Y, Gill KS (2001). Flow cytometric sorting of maize chromosome 9 from an oat–maize chromosome addition line. Theor Appl Genet.

[CR121] Li L, Arumuganathan K, Gill KS, Song Y (2004). Flow sorting and microcloning of maize chromosome 1. Hereditas.

[CR122] Lichten M, de Massy B (2011). The impressionistic landscape of meiotic recombination. Cell.

[CR123] Lindenbaum M, Perkins E, Csonka E, Fleming E, Garcia L, Greene A, Gung L, Hadlaczky G, Lee E, Leung J, MacDonald N, Maxwell A, Mills K, Monteith D, Perez CF, Shellard J, Stewart S, Stodola T, Vandenborre D, Vanderbyl S, Ledebur HC (2004). A mammalian artificial chromosome engineering system (ACE System) applicable to biopharmaceutical protein production, transgenesis and gene-based cell therapy. Nucleic Acids Res.

[CR124] Lucretti S, Doležel J (1997). Bivariate flow karyotyping in broad bean (*Vicia faba*). Cytometry.

[CR125] Lucretti S, Doležel J, Schubert I, Fuchs J (1993). Flow karyotyping and sorting of *Vicia faba* chromosomes. Theor Appl Genet.

[CR126] Luo MC, Thomas C, You FM, Hsiao J, Shu OY, Buell CR, Malandro M, McGuire PE, Anderson OD, Dvorak J (2003). High-throughput fingerprinting of bacterial artificial chromosomes using the SNaPshot labeling kit and sizing of restriction fragments by capillary electrophoresis. Genomics.

[CR127] Lysák MA, Číhalíková J, Kubaláková M, Šimková H, Künzel G, Doležel J (1999). Flow karyotyping and sorting of mitotic chromosomes of barley (*Hordeum vulgare* L.). Chromosom Res.

[CR128] Lysák MA, Fransz PF, Ali HBM, Schubert I (2001). Chromosome painting in *Arabidopsis thaliana*. Plant J.

[CR129] Ma RZ, Russ I, Park C, Heyen DW, Beever JE, Green CA, Lewin HA (1996). Isolation and characterization of 45 polymorphic microsatellites from the bovine genome. Anim Genet.

[CR130] Ma YZ, Lee JH, Li LC, Uchiyama S, Ohmido N, Fukui K (2005). Fluorescent labeling of plant chromosomes in suspension by FISH. Gene Genet Syst.

[CR131] Ma L, Xiao Y, Huang H, Wang QW, Rao WN, Feng Y, Zhang K, Song Q (2010). Direct determination of molecular haplotypes by chromosome microdissection. Nat Methods.

[CR132] Macas J, Doležel J, Lucretti S, Pich U, Meister A, Fuchs J, Schubert I (1993). Localization of seed protein genes on flow-sorted field bean chromosomes. Chromosom Res.

[CR133] Macas J, Doležel J, Gualberti G, Pich U, Schubert I, Lucretti S (1995). Primer-induced labelling of pea and field bean chromosomes in situ and in suspension. Biotechniques.

[CR134] Macas J, Gualberti G, Nouzová M, Samec P, Lucretti S, Doležel J (1996). Construction of chromosome-specific DNA libraries covering the whole genome of field bean (*Vicia faba* L.). Chromosom Res.

[CR135] Macnab S, Whitehouse A (2009). Progress and prospects: human artificial chromosomes. Gene Ther.

[CR136] Mardis ER (2008). Next-generation DNA sequencing methods. Annu Rev Genom Hum Genet.

[CR137] Margueron R, Reinberg D (2010). Chromatin structure and the inheritance of epigenetic information. Nat Rev Genet.

[CR138] Matsson P, Rydberg B (1981). Analysis of chromosomes from human peripheral lymphocytes by flow cytometry. Cytometry.

[CR139] Matsunaga S, Kawano S, Michimoto T, Higashiyama T, Nakao S, Sakai A, Kuroiwa T (1999). Semi-automatic laser beam microdissection of the Y chromosome and analysis of Y chromosome DNA in a dioecious plant, *Silene latifolia*. Plant Cell Physiol.

[CR140] Mayer KFX, Taudien S, Martis M, Šimková H, Suchánková P, Gundlach H, Wicker T, Petzold A, Felder M, Steuernagel B, Scholz U, Graner A, Platzer M, Doležel J, Stein N (2009). Gene content and virtual gene order of barley chromosome 1 H. Plant Physiol.

[CR141] Mayer KFX, Martis M, Hedley PE, Simkova H, Liu H, Morris JA, Steuernagel B, Taudien S, Roessner S, Gundlach H, Kubalakova M, Suchankova P, Murat F, Felder M, Nussbaumer T, Graner A, Salse J, Endo T, Sakai H, Tanaka T, Itoh T, Sato K, Platzer M, Matsumoto T, Scholz U, Doležel J, Waugh R, Stein N (2011). Unlocking the barley genome by chromosomal and comparative genomics. Plant Cell.

[CR142] McCormick MK, Buckler A, Bruno W, Campbell E, Shera K, Torney D, Deaven L, Moyzis R (1993). Construction and characterization of a YAC library with a low frequency of chimeric clones from flow-sorted human chromosome 9. Genomics.

[CR143] McCormick MK, Campbell E, Deaven L, Moyzis R (1993). Low-frequency chimeric yeast artificial chromosome libraries from flow-sorted human chromosomes 16 and 21. Proc Natl Acad Sci USA.

[CR144] Meksem K, Kahl G (2005). The handbook of plant genome mapping. Genetic and physical mapping.

[CR145] Metzker ML (2010). Sequencing technologies—the next generation. Nat Rev Genet.

[CR146] Miller JR, Dixon SC, Miller NGA, Tucker EM, Hindkjaer J, Thomsen PD (1992). A chromosome-1-specific DNA library from the domestic pig (*Sus scrofa domestica*). Cytogenet Cell Genet.

[CR147] Molnár I, Kubaláková M, Šimková H, Cseh A, Molnár-Láng M, Doležel J (2011). Chromosome isolation by flow sorting in *Aegilops umbellulata* and *Ae. comosa* and their allotetraploid hybrids *Ae. biuncialis* and *Ae. geniculata*. PLoS One.

[CR148] Muñoz-Amatriaín M, Moscou MJ, Bhat PR, Svensson JT, Bartoš J, Suchánková P, Šimková H, Endo TR, Fenton RD, Lonardi S, Castillo AM, Chao S, Cistué L, Cuesta-Marcos A, Forrest KL, Hayden MJ, Hayes PM, Horsley RD, Makoto K, Moody D, Sato K, Vallés MP, Wulff BBH, Muehlbauer GJ, Doležel J, Close TJ (2011). An improved consensus linkage map of barley based on flow-sorted chromosomes and single nucleotide polymorphism markers. Plant Genome.

[CR149] Neely RK, Deen J, Hofkens J (2011). Optical mapping of DNA: single-molecule-based methods for mapping genomes. Biopolymers.

[CR150] Neumann P, Lysák M, Doležel J, Macas J (1998). Isolation of chromosomes from *Pisum sativum* L. hairy root cultures and their analysis by flow cytometry. Plant Sci.

[CR151] Neumann P, Požárková D, Vrána J, Doležel J, Macas J (2002). Chromosome sorting and PCR-based physical mapping in pea (*Pisum sativum* L.). Chromosom Res.

[CR152] Ng BL, Carter NP (2006). Factors affecting flow karyotype resolution. Cytometry A.

[CR153] Ng BL, Yang FY, Carter NP (2007). Flow analysis and sorting of microchromosomes (< 3 Mb). Cytometry A.

[CR154] Nie W, O’Brien PCM, Ng BL, Fu B, Volobouev V, Carter NP, Ferguson-Smith MA, Yang F (2009). Avian comparative genomics: reciprocal chromosome painting between domestic chicken (*Gallus gallus*) and the stone curlew (*Burhinus oedicnemus*, Charadriiformes)—an atypical species with low diploid number. Chromosom Res.

[CR155] Nie W, Wang J, Su W, Wang D, Tanomtong A, Perelman PL, Graphodatsky AS, Yang F (2012). Chromosomal rearrangements and karyotype evolution in carnivores revealed by chromosome painting. Heredity.

[CR156] Nizetic D, Gellen L, Hamvas R, Mott R, Grigoriev A, Vatcheva R, Zehetner G, Yaspo ML, Dutriau A, Lopes C, Delabar J-M, Van Broeckhoven C, Potler M-C, Lehrach H (1994). An integrated YAC-overlap and “cosmid pocket” map of the human chromosome 21. Hum Mol Genet.

[CR157] Nusse M, Viaggi S, Bonatti S (1992). Identification and fate of a marker chromosome in methotrexate-resistant V79, B7 cells by flow karyotyping and sorting, metaphase analysis and in situ hybridization. Anal Cell Pathol.

[CR158] Osman K, Higgins JD, Sanchez-Moran E, Armstrong SJ, Franklin FCH (2011). Pathways to meiotic recombination in *Arabidopsis thaliana*. New Phytol.

[CR159] Paux E, Roger D, Badaeva E, Gay G, Bernard M, Sourdille P, Feuillet C (2006). Characterizing the composition and evolution of homoeologous genomes in hexaploid wheat through BAC-end sequencing on chromosome 3B. Plant J.

[CR160] Paux E, Sourdille P, Salse J, Saintenac C, Choulet F, Leroy P, Korol A, Michalak M, Kianian S, Spielmeyer W, Lagudah E, Somers D, Kilian A, Alaux M, Vautrin S, Bergès H, Eversole K, Appels R, Šafář J, Šimková H, Doležel J, Bernard M, Feuillet C (2008). A physical map of the 1-gigabase bread wheat chromosome 3B. Science.

[CR161] Pawlowski WP (2010). Chromosome organization and dynamics in plants. Curr Opin Plant Biol.

[CR162] Pich U, Meister A, Macas J, Doležel J, Lucretti S, Schubert I (1995). Primed in situ labelling facilitates flow sorting of similar sized chromosomes. Plant J.

[CR163] Pinkel D, Segraves R, Sudar D, Clark S, Poole I, Kowbel D, Collins C, Kuo W-L, Chen C, Zhai Y, Dairkee SH, Ljung B-M, Gray JW, Albertson DG (1998). High resolution analysis of DNA copy number variation using comparative genomic hybridization to microarrays. Nat Genet.

[CR164] Požárková D, Koblížková A, Román B, Torres AM, Lucretti S, Lysák M, Doležel J, Macas J (2002). Development and characterization of microsatellite markers from chromosome 1-specific DNA libraries of *Vicia faba*. Biol Plant.

[CR165] Rens W, Vanoven CH, Stap J, Jakobs ME, Aten JA (1994). Slit-scanning technique using standard cell sorter instruments for analyzing and sorting nonacrocentric human-chromosomes, including small ones. Cytometry.

[CR166] Rommel B, Hutter KJ, Bullerdiek J, Barnitzke S, Goerttler K, Schloot W (1988). Identification of flow-sorted chromosomes by G-banding and in situ hybridization. Cytometry.

[CR167] Rubeš J, Pinton A, Bonnet-Garnier A, Fillon V, Musilová P, Michalová K, Kubíčková S, Ducos A, Yerle M (2009). Fluorescence in situ hybridization applied to domestic animal cytogenetics. Cytogenet Genome Res.

[CR168] Šafář J, Bartoš J, Janda J, Bellec A, Kubaláková M, Valárik M, Pateyron S, Weiserová J, Tušková R, Číhalíková J, Vrána J, Šimková H, Faivre-Rampant P, Sourdille P, Caboche M, Bernard M, Doležel J, Chalhoub B (2004). Dissecting large and complex genomes: flow sorting and BAC cloning of individual chromosomes from bread wheat. Plant J.

[CR169] Šafář J, Šimková H, Kubaláková M, Číhalíková J, Suchánková P, Bartoš J, Doležel J (2010). Development of chromosome-specific BAC resources for genomics of bread wheat. Cytogenet Genome Res.

[CR170] Sankovic N, Delbridge ML, Grützner F, Ferguson-Smith MA, O’Brien PCM, Marshall Graves JA (2006). Construction of a highly enriched marsupial Y chromosome-specific BAC sub-library using isolated Y chromosomes. Chromosom Res.

[CR171] Sargan DR, Yang FT, Squire M, Milne BS, O’Brien PCM, Ferguson-Smith MA (2000). Use of flow-sorted canine chromosomes in the assignment of canine linkage, radiation hybrid, and syntenic groups to chromosomes: refinement and verification of the comparative chromosome map for dog and human. Genomics.

[CR172] Scalenghe F, Turco E, Ederström JE, Pirrotta V, Melli M (1981). Microdissection and cloning of DNA from a specific region of *Drosophila melanogaster* polytene chromosomes. Chromosoma.

[CR173] Scherthan H, Cremer T, Arnason U, Weier HU, Limadefaria A, Fronicke L (1994). Comparative chromosome painting discloses homologous segments in distantly related mammals. Nat Genet.

[CR174] Schmitz A, Oustry A, Chaput B, Bahridarwich I, Yerle M, Millan D, Frelat G, Cribiu EP (1995). The bovine bivariate flow karyotype and peak identification by chromosome painting with PCR-generated probes. Mamm Genome.

[CR175] Schondelmaier J, Martin R, Jahoor A, Houben A, Graner A, Koop HU, Herrmann RG, Jung C (1993). Microdissection and microcloning of the barley (*Hordeum vulgare* L.) chromosome 1HS. Theor Appl Genet.

[CR176] Schröck E, du Manoir S, Veldman T, Schoell B, Wienberg J, Ferguson-Smith MA, Ning Y, Ledbetter DH, Bar-Am I, Soenksen D, Garini Y, Ried T (1996). Multicolor spectral karyotyping of human chromosomes. Science.

[CR177] Schubert I, Oud JL (1997). There is an upper limit of chromosome size for normal development of an organism. Cell.

[CR178] Schubert I, Doležel J, Houben A, Scherthan H, Wanner G (1993). Refined examination of plant metaphase chromosome structure at different levels made feasible by new isolation methods. Chromosoma.

[CR179] Schubert I, Fransz PF, Fuchs J, de Jong JH (2001). Chromosome painting in plants. Methods Cell Sci.

[CR180] Schwarzacher T, Wang ML, Leitch AR, Miller N, Moore G, Heslop-Harrison JS (1997). Flow cytometric analysis of the chromosomes and stability of a wheat cell-culture line. Theor Appl Genet.

[CR181] Shepel LA, Morrissey LW, Hsu LC, Gould MN (1994). Bivariate flow karyotyping, sorting, and peak assignment of all rat chromosomes. Genomics.

[CR182] Shepel LA, Lan H, Brasic GM, Gheen ME, Hsu LC, Haag JD, Gould MN (1998). Mapping of 55 new rat microsatellite markers from chromosome-specific libraries. Mamm Genome.

[CR183] Sillar R, Young BD (1981). A new method for the preparation of metaphase chromosomes for flow analysis. J Histochem Cytochem.

[CR184] Silverman GA, Schneider SS, Massa HF, Flint A, Lalande M, Leonard JC, Overhauser J, van den Engh G, Trask BJ (1995). The 18Q(−) syndrome—analysis of chromosomes by bivariate flow karyotyping and the PCR reveals a successive set of deletion breakpoints within 18Q21.2–Q22.2. Am J Hum Genet.

[CR185] Šimková H, Šafář J, Suchánková P, Kovářová P, Bartoš J, Kubaláková M, Janda J, Číhalíková J, Mago R, Lelley T, Doležel J (2008). A novel resource for genomics of Triticeae: BAC library specific for the short arm of rye (*Secale cereale* L.) chromosome 1R (1RS). BMC Genomics.

[CR186] Speicher MR, Gwyn Ballard S, Ward DC (1996). Karyotyping human chromosomes by combinatorial multi-fluor FISH. Nat Genet.

[CR187] Stallings RL, Torney DC, Hildebrand CE, Longmire JL, Deaven LL, Jett JH, Doggett NA, Moyzis RK (1990). Physical mapping of human chromosomes by repetitive sequence fingerprinting. Proc Natl Acad Sci USA.

[CR188] Stepanov SI, Konyshev VN, Kotlovanova LV, Roganov AP (1996). Karyotyping of individual cells with flow cytometry. Cytometry.

[CR189] Stubblefield E, Oro J (1982). The isolation of specific chicken macrochromosomes by zonal centrifugation and flow sorting. Cytometry.

[CR190] Stubblefield E, Cram S, Deaven L (1975). Flow microfluorometric analysis of isolated Chinese-hamster chromosomes. Exp Cell Res.

[CR191] Suchánková P, Kubaláková M, Kovářová P, Bartoš J, Číhalíková J, Molnár-Láng M, Endo TR, Doležel J (2006). Dissection of the nuclear genome of barley by chromosome flow sorting. Theor Appl Genet.

[CR192] Sudbery I, Stalker J, Simpson JT, Keane T, Rust AG, Hurles ME, Walter K, Lynch D, Teboul L, Brown SD, Li H, Ning Z, Nadeau JH, Croniger CM, Durbin R, Adams DJ (2009). Deep short-read sequencing of chromosome 17 from the mouse strains A/J and CAST/Ei identifies significant germline variation and candidate genes that regulate liver triglyceride levels. Genome Biol.

[CR193] Szinay D, Bai Y, Visser R, de Jong H (2010). FISH applications for genomics and plant breeding strategies in tomato and other solanaceous crops. Cytogenet Genome Res.

[CR194] Takata H, Uchiyama S, Nakamura N, Nakashima S, Kobayashi S, Sone T, Kimura S, Lahmers S, Granzier H, Labeit S, Matsunaga S, Fukui K (2007). A comparative proteome analysis of human metaphase chromosomes isolated from two different cell lines reveals a set of conserved chromosome-associated proteins. Gene Cell.

[CR195] Teague B, Waterman MS, Goldstein S, Potamousis K, Zhou SG, Reslewic S, Sarkar D, Valouev A, Churas C, Kidd JM, Kohn S, Runnheim R, Lamers C, Forrest D, Newton MA, Eichler EE, Kent-First M, Surti U, Livny M, Schwartz DC (2010). High-resolution human genome structure by single-molecule analysis. Proc Natl Acad Sci USA.

[CR196] Telenius H, Pelmear AH, Tunnacliffe A, Carter NP, Behmel A, Ferguson-Smith MA, Nordenskjöld M, Pfragner R, Ponder BA (1992). Cytogenetic analysis by chromosome painting using DOP-PCR amplified flow-sorted chromosomes. Gene Chromosome Cancer.

[CR197] Telenius H, Devos D, Blennow E, Willat LR, Ponder BAJ, Carter NP (1993). Chromatid contamination can impair the purity of flow-sorted metaphase chromosomes. Cytometry.

[CR198] ten Hoopen R, Manteuffel R, Doležel J, Malysheva L, Schubert I (2000). Evolutionary conservation of kinetochore protein sequences in plants. Chromosoma.

[CR199] Trask BJ, van den Engh G, Gray J, van der Laan M, Turner B (1984). Immunofluorescent detection of histone-2B on metaphase chromosomes using flow-cytometry. Chromosoma.

[CR200] Trask BJ, Van den Engh G, Landegent J, In de Wal NJ, Van der Ploegh M (1985). Detection of DNA sequences in nuclei in suspension by in situ hybridization and dual beam flow cytometry. Science.

[CR201] Trask BJ, Mefford H, van den Engh G, Massa HF, Juyal RC, Potocki L, Finucane B, Abuelo DN, Witt DR, Magenis E, Baldini A, Greenberg F, Lupski JR, Patel PI (1996). Quantification by flow cytometry of chromosome-17 deletions in Smith–Magenis syndrome patients. Hum Genet.

[CR202] Treangen TJ, Salzberg SL (2012). Repetitive DNA and next-generation sequencing: computational challenges and solutions. Nat Rev Genet.

[CR203] Überall I, Vrána J, Bartoš J, Šmerda J, Doležel J, Havel L (2004). Isolation of chromosomes from *Picea abies* L. and their analysis by flow cytometry. Biol Plant.

[CR204] Uchiyama S, Kobayashi S, Takata H, Ishihara T, Hori N, Higashi T, Hayashihara K, Sone T, Higo D, Nirasawa T, Takao T, Matsunaga S, Fukui K (2005). Proteome analysis of human metaphase chromosomes. J Biol Chem.

[CR205] Valárik M, Bartoš J, Kovářová P, Kubaláková M, de Jong H, Doležel J (2004). High-resolution FISH on super-stretched flow-sorted plant chromosomes. Plant J.

[CR206] van den Engh G, Trask B, Cram S, Bartholdi M (1984). Preparation of chromosome suspensions for flow-cytometry. Cytometry.

[CR207] Van Dilla MA, Deaven LL (1990). Construction of gene libraries for each human chromosome. Cytometry.

[CR208] Van Dilla MA, Deaven LL, Albright KL, Allen NA, Aubuchon MR, Bartholdi MF, Browne NC, Campbell EW, Carrano AV, Clark LM, Cram LS, Fuscoe JC, Gray JW, Hildebrand CE, Jackson PJ, Jett JH, Longmire JL, Lozes CR, Luedemann ML, Martin JC, McNinch JS, Meincke LJ, Mendelsohn ML, Meyne J, Moyzis RK, Munk AC, Perlman J, Peters DC, Silva AJ, Trask BJ (1986). Human chromosome-specific DNA libraries: construction and availability. Biotechnology.

[CR209] Van Devanter DR, Choongkittaworn NM, Dyer KA, Aten J, Otto P, Behler C, Bryant EM, Rabinovitch PS (1994). Pure chromosome-specific PCR libraries from single sorted chromosomes. Proc Natl Acad Sci USA.

[CR210] Veltman IM, Veltman JA, Arkesteijn G, Janssen IM, Vissers LE, de Jong PJ, van Kessel AG, Schoenmakers EF (2003). Chromosomal breakpoint mapping by arrayCGH using flow-sorted chromosomes. Biotechniques.

[CR211] Verdaasdonk JS, Bloom K (2011). Centromeres: unique chromatin structures that drive chromosome segregation. Nat Rev Mol Cell Biol.

[CR212] Veuskens J, Marie D, Brown SC, Jacobs M, Negrutiu I (1995). Flow sorting of the Y sex chromosome in the dioecious plant *Melandrium album*. Cytometry.

[CR213] Vitharana SN, Wilson GS (2006). Fractionation of chromosome 15 with an affinity-based approach using magnetic beads. Genomics.

[CR214] Vitulo N, Albiero A, Forcato C, Campagna D, Dal Pero F, Bagnaresi P, Colaiacovo M, Faccioli P, Lamontanara A, Šimková H, Kubaláková M, Perrotta G, Facella P, Lopez L, Pietrella M, Gianese G, Doležel J, Giuliano G, Cattivelli L, Valle G, Stanca AM (2011). First survey of the wheat chromosome 5A composition through a next generation sequencing approach. PLoS One.

[CR215] Vláčilová K, Ohri D, Vrána J, Číhalíková J, Kubaláková M, Kahl G, Doležel J (2002). Development of flow cytogenetics and physical genome mapping in chickpea (*Cicer arietinum* L.). Chromosom Res.

[CR216] Vooijs M, Yu LC, Tkachuk D, Pinkel D, Johnson D, Gray JW (1993). Libraries for each human-chromosome, constructed from sorter-enriched chromosomes by using linker-adapter PCR. Am J Hum Genet.

[CR217] Vrána J, Kubaláková M, Šimková H, Číhalíková J, Lysák MA, Doležel J (2000). Flow-sorting of mitotic chromosomes in common wheat (*Triticum aestivum* L*.*). Genetics.

[CR218] Wang ML, Leitch AR, Schwarzacher T, Heslop-Harrison JS, Moore G (1992). Construction of a chromosome-enriched *Hpa*II library from flow-sorted wheat chromosomes. Nucleic Acids Res.

[CR219] Wanner G, Formanek H, Martin R, Herrmann RG (1991). High resolution scanning electron microscopy of plant chromosomes. Chromosoma.

[CR220] Wei FS, Zhang JW, Zhou SG, He RF, Schaeffer M, Collura K, Kudrna D, Faga BP, Wissotski M, Golser W, Rock SM, Graves TA, Fulton RS, Coe E, Schnable PS, Schwartz DC, Ware D, Clifton SW, Wilson RK, Wing RA (2009). The physical and genetic framework of the maize B73 genome. PLoS Genet.

[CR221] Wenzl P, Suchánková P, Carling J, Šimková H, Huttner E, Kubaláková M, Sourdille P, Paul E, Feuillet C, Kilian A, Doležel J (2010). Isolated chromosomes as a new and efficient source of DArT markers for the saturation of genetic maps. Theor Appl Genet.

[CR222] Wicker T, Mayer KFX, Gundlach H, Martis M, Steuernagel B, Scholz U, Šimková H, Kubaláková M, Choulet F, Taudien S, Platzer M, Feuillet C, Fahima T, Budak H, Doležel J, Keller B, Stein N (2011). Frequent gene movement and pseudogene evolution is common to the large and complex genomes of wheat, barley, and their relatives. Plant Cell.

[CR223] Wlodkowic D, Darzynkiewicz Z (2011) Rise of the micromachines: microfluidics and the future of cytometry. In: Darzynkiewicz Z, Holden E, Orfao A, Telford W., Wlodkowic (eds) Methods in cell biology, vol 102. Recent advances in cytometry, part A: instrumentation, methods. Academic Press, San Diego, pp 105–125 10.1016/B978-0-12-374912-3.00005-5 PMC3241275 21704837

[CR224] Yang H, Chen X, Wong WH (2011). Completely phased genome sequencing through chromosome sorting. Proc Natl Acad Sci USA.

[CR225] Yanowitz J (2010). Meiosis: making a break for it. Curr Opin Cell Biol.

[CR226] Yerle M, Schmitz A, Milan D, Chaput B, Monteagudo L, Vaiman M, Frelat G, Gellin J (1993). Accurate characterization of porcine bivariate flow karyotype by PCR and fluorescence in situ hybridization. Genomics.

[CR227] Young BD, Ferguson-Smith MA, Sillar R, Boyd E (1981). High-resolution analysis of human peripheral lymphocyte chromosomes by flow-cytometry. Proc Natl Acad Sci USA.

[CR228] Young ND, Debellé F, Oldroyd GE, Geurts R, Cannon SB, Udvardi MK, Benedito VA, Mayer KF, Gouzy J, Schoof H, Van de Peer Y, Proost S, Cook DR, Meyers BC, Spannagl M, Cheung F, De Mita S, Krishnakumar V, Gundlach H, Zhou S, Mudge J, Bharti AK, Murray JD, Naoumkina MA, Rosen B, Silverstein KA, Tang H, Rombauts S, Zhao PX, Zhou P, Barbe V, Bardou P, Bechner M, Bellec A, Berger A, Bergès H, Bidwell S, Bisseling T, Choisne N, Couloux A, Denny R, Deshpande S, Dai X, Doyle JJ, Dudez AM, Farmer AD, Fouteau S, Franken C, Gibelin C, Gish J, Goldstein S, González AJ, Green PJ, Hallab A, Hartog M, Hua A, Humphray SJ, Jeong DH, Jing Y, Jöcker A, Kenton SM, Kim DJ, Klee K, Lai H, Lang C, Lin S, Macmil SL, Magdelenat G, Matthews L, McCorrison J, Monaghan EL, Mun JH, Najar FZ, Nicholson C, Noirot C, O’Bleness M, Paule CR, Poulain J, Prion F, Qin B, Qu C, Retzel EF, Riddle C, Sallet E, Samain S, Samson N, Sanders I, Saurat O, Scarpelli C, Schiex T, Segurens B, Severin AJ, Sherrier DJ, Shi R, Sims S, Singer SR, Sinharoy S, Sterck L, Viollet A, Wang BB, Wang K, Wang M, Wang X, Warfsmann J, Weissenbach J, White DD, White JD, Wiley GB, Wincker P, Xing Y, Yang L, Yao Z, Ying F, Zhai J, Zhou L, Zuber A, Dénarié J, Dixon RA, May GD, Schwartz DC, Rogers J, Quétier F, Town CD, Roe BA (2011). The *Medicago* genome provides insight into the evolution of rhizobial symbioses. Nature.

[CR229] Zatloukalová P, Hřibová E, Kubaláková M, Suchánková P, Šimková H, Adoración C, Kahl G, Millán T, Doležel J (2011). Integration of genetic and physical maps of the chickpea (*Cicer arietinum* L.) genome using flow-sorted chromosomes. Chromosom Res.

[CR230] Zhou SG, Wei FS, Nguyen J, Bechner M, Potamousis K, Goldstein S, Pape L, Mehan MR, Churas C, Pasternak S, Forrest DK, Wise R, Ware D, Wing RA, Waterman MS, Livny M, Schwartz DC (2009). A single molecule scaffold for the maize genome. PLoS Genet.

[CR231] Zhou VW, Goren A, Bernstein BE (2011). Charting histone modifications and the functional organization of mammalian genomes. Nat Rev Genet.

